# Multifunctional elastin-like polypeptide nanocarriers for efficient miRNA delivery in cancer therapy

**DOI:** 10.1186/s12951-024-02559-5

**Published:** 2024-05-27

**Authors:** Jisan Hong, Dahye Sim, Byung-Heon Lee, Vijaya Sarangthem, Rang-Woon Park

**Affiliations:** Department of Biochemistry and Cell Biology, Cell & Matrix Research Institute, Kyungpook National University, School of Medicine, Daegu, 41944 Republic of Korea

**Keywords:** miRNA-34a, Tumor targeting, ELP nanoparticle, IL-4 receptor, Cell penetrating peptide, Apoptosis, 3D spheroid, Tumor inhibition

## Abstract

**Background:**

The exogenous delivery of miRNA to mimic and restore miRNA-34a activity in various cancer models holds significant promise in cancer treatment. Nevertheless, its effectiveness is often impeded by challenges, including a short half-life, propensity for off-target accumulation, susceptibility to inactivation by blood-based enzymes, concerns regarding patient safety, and the substantial cost associated with scaling up. As a means of overcoming these barriers, we propose the development of miRNA-loaded Tat-A86 nanoparticles by virtue of Tat-A86's ability to shield the loaded agent from external environmental factors, reducing degradation and inactivation, while enhancing circulation time and targeted accumulation.

**Results:**

Genetically engineered Tat-A86, featuring 16 copies of the interleukin-4 receptor (IL-4R)-binding peptide (AP1), Tat for tumor penetration, and an elastin-like polypeptide (ELP) for presenting target ligands and ensuring stability, served as the basis for this delivery system. Comparative groups, including Tat-E60 and A86, were employed to discern differences in binding and penetration. The designed ELP-based nanoparticle Tat-A86 effectively condensed miRNA, forming stable nanocomplexes under physiological conditions. The miRNA/Tat-A86 formulation bound specifically to tumor cells and facilitated stable miRNA delivery into them, effectively inhibiting tumor growth. The efficacy of miRNA/Tat-A86 was further evaluated using three-dimensional spheroids of lewis lung carcinoma (LLC) as in vitro model and LLC tumor-bearing mice as an in vivo model. It was found that miRNA/Tat-A86 facilitates effective cell killing by markedly improving miRNA penetration, leading to a substantial reduction in the size of LLC spheroids. Compared to other controls, Tat-A86 demonstrated superior efficacy in suppressing the growth of 3D cellular aggregates. Moreover, at equivalent doses, miRNA-34a delivered by Tat-A86 inhibited the growth of LLC cells in allograft mice.

**Conclusions:**

Overall, these studies demonstrate that Tat-A86 nanoparticles can deliver miRNA systemically, overcoming the basic hurdles impeding miRNA delivery by facilitating both miRNA uptake and stability, ultimately leading to improved therapeutic effects.

**Graphical Abstract:**

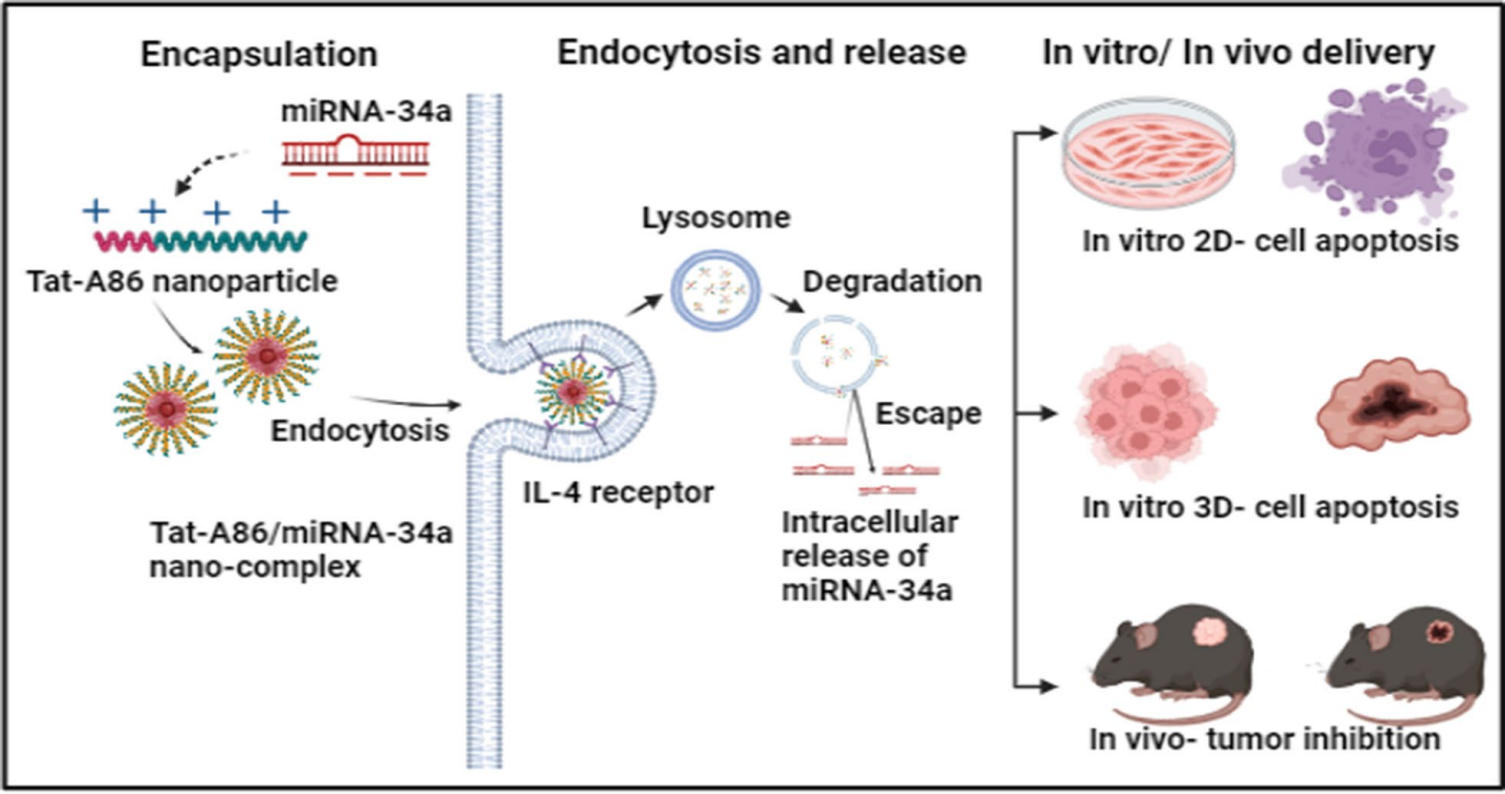

**Supplementary Information:**

The online version contains supplementary material available at 10.1186/s12951-024-02559-5.

## Introduction

MicroRNAs (miRNAs) have gained prominence in cancer therapy because of their applications in cancer prognosis, pathogenesis, diagnosis, and treatment. Comprising approximately 22 nucleotides in length, miRNAs are small, highly conserved, and non-protein coding molecules [1] that regulate gene expression, influencing processes like cell growth, differentiation, and death [2]. In cancer therapy, certain miRNAs can function either as tumor suppressors or oncogenes, and their targeted delivery to cancer cells can yield therapeutic benefits [3]. The miR-34 family, along with the let-7 and miR-200 families, collectively constitute the three major tumor-suppressive miRNA families. Downregulation or loss of expression of miR-34a is linked to various cancers, including glioblastomas and malignant peripheral nerve sheath tumors, as well as breast, colon, ovarian, pancreatic, and prostate cancers [4–10]. Evidence indicates that miR-34a directly targets the 3' UTRs of diverse oncogenic mRNAs, including Bcl-2, SIRT1, Fra-1, E2F, c-Met, Notch1, Notch2, CDK4/6, VEGF, ARAF, PIK3R2, cyclin D3, cyclin E2, and PLK1 [6, 7, 10–15], which may explain its tumor-suppressing properties. Moreover, low miR-34a expression correlates with larger tumor size [16]. Recent breakthroughs in the field of miR-34a biology have sparked significant enthusiasm among biopharmaceutical companies.

MiRNA replacement therapy, which involves reintroducing miRNAs into cancer cells to restore their normal function, has been widely assessed in preclinical trials [17, 18]. Particularly, microRNA-34a (or miR-34a) has been extensively investigated as a potential candidate for lung cancer therapy [19]. Restoring miRNA-34a levels in pancreatic cancer promoted the downregulation of Bcl-2 and Notch 1/2 expression, impeding cell growth and invasion [20]. Furthermore, the decreased expression of miRNA-34a implicated both the development and progression of epithelial ovarian cancer [5]. Notably, enforced miRNA-34a expression inhibited cell growth and promoted apoptosis in p53-mutant gastric cancer cells [21]. However, despite evidence of the potential anti-cancer effects of miRNAs, their clinical application is limited by practical challenges. The primary concern when developing miRNA as new drugs is ensuring their safety and effectiveness. The key obstacles associated with miRNA delivery include challenges in accessing the target cells and the risk of off-target effects. Moreover, naked miRNA is ineffective due to its poor stability, short half-life, and susceptibility to degradation or inactivation by nucleic acid-degrading enzymes [22, 23]. Therefore, harnessing the therapeutic effects of miRNAs necessitates the development of optimal delivery systems, such as nanoparticles, to encapsulate and protect miRNAs until they reach their target cells within tumors.

Currently, both non-viral and viral delivery systems are employed for effective miRNA delivery. Non-viral systems utilize organic, inorganic, or polymer-based carriers for miRNA delivery, whereas virus-based systems employ lentiviruses, retroviruses, adenoviruses, and adeno-associated viruses [24, 25]. While viral vectors exhibit notable transfection efficiency, their application is hindered by immunogenicity and cytotoxicity issues. In contrast, non-viral delivery systems are usually less toxic and immunogenic; however, they exhibit lower transfection efficiency. Various synthetic polymers, including poly(ethylene imine)s (PEIs) [26, 27], PLGA [28, 29], poly(ε-caprolactone) (PCL) [30], and polyurethanes (PUs) [31, 32], have been employed for miRNA delivery. However, their application in cancer therapeutics is constrained by issues such as high cellular toxicity, inadequate encapsulation, and non-specific targeting effects [33–35]. Several researchers have attempted to overcome these limitations by modifying nanoparticle surfaces with ligands that bind to receptors on target cells. This modification facilitates receptor-mediated endocytosis of nanoparticles, consequently improving the distribution and localization of miRNAs within tissues [33, 36]. Nevertheless, the use of polymers is often limited because specialized techniques are required for modifying block polymers with targeting peptides. Moreover, controlling their physicochemical properties, such as molecular weight (MW) and polydispersity, is a complex endeavor [37]. Thus, developing novel solutions to address these challenges is imperative.

Here, we demonstrate that multifunctional elastin-like polypeptide (ELP)-based nanoparticles are an effective solution to overcome the aforementioned challenges and can efficiently deliver miRNA to cancer cells, potentially regulating gene expression and inhibiting tumour growth. This approach has the potential to improve the specificity and efficacy of cancer treatment while simultaneously minimizing the side effects on healthy tissues. Numerous investigations have meticulously expounded upon the merits of incorporating ELPs in gene delivery systems [33, 38]. ELPs, derived from the hydrophobic domain of tropoelastin, are composed of pentapeptide (Val–Pro–Gly–Xaa–Gly) repeats, where the 'guest residue' Xaa encompasses any amino acid except proline [39, 40]. Thus, ELPs are non-toxic, non-immunogenic, and biodegradable; these features make them apt for gene delivery applications [41]. Genetically encoded synthesis offers exceptional control over the size, shape, and physical attributes of ELP nanoparticles, enabling the incorporation of peptide or protein domains designed for specific targeting [42]. Additionally, the temperature-responsive behavior of ELP nanoparticles facilitates their phase transition in aqueous solutions when heated above their inverse transition temperature (Tt), followed by complete resolubilization below their Tt [43]. This reversible phase transition property of ELPs ensures both easy purification and a high yield through the inverse transition cycling (ITC) method. Thus, ELP-based drug delivery systems have the potential to facilitate cost-effective, efficient, and precise miRNA delivery.

In a previous study, we demonstrated the potential of micelle-forming multivalent targeting based AP1-ELP (A86) polymers, emphasizing their heightened binding activity, rapid cellular entry, and efficient processing within the interleukin-4 receptor (IL-4R)-dependent endocytic pathway across diverse cancer cell lines [44]. Exclusively, A86 exhibited superior tumor penetration and substantial retention within tumor tissues while also mitigating non-specific accumulation in critical organs in a murine model with 4T1 allografted tumors. In the present study, we genetically synthesized Tat-A86 nanoparticle-based delivery systems, aiming to improve in vivo delivery, prevent degradation, and augment the target specificity of miRNA-34a. We comprehensively elucidated the mechanistic intricacies of miRNA delivery, including encapsulation efficiency, ligand-specific uptake, cellular entry, lysosomal escape, the interaction between miRNA and its intracellular target, and tumor inhibition. Furthermore, the cellular uptake, tumor penetration, and antitumor activity of the miRNA/ELP nanoformulations were evaluated using three-dimensional (3D) spheroids and Lewis lung carcinoma (LLC)-bearing mice as the in vitro and in vivo models, respectively.

## Materials and methods

### Protein purification

*Escherichia coli* BL21 competent cells were individually transformed with pET 25b + vectors carrying the Tat-E60, A86, and Tat-A86 genes. To initiate the culture, a single colony was inoculated into 10 mL of Luria–Bertani (LB) broth containing 0.1 mg of ampicillin and incubated overnight at 37 °C in a shaking incubator. The resulting starter culture was then transferred into 700 mL of fresh terrific broth media supplemented with ampicillin and incubated in a shaking incubator until its absorbance at 600 nm reached a value between 0.8 and 1.0. To induce protein synthesis, 1 mM of isopropyl β- d-1-thiogalactopyranoside (IPTG; Carbosynth Limited, Berkshire, UK) was added to the culture and incubated for 4 h. Subsequently, the culture was centrifuged at 4000 rpm for 20 min at 4 °C and the pellet obtained was suspended in 3 mL of phosphate-buffered saline (PBS). Then, 20 mL of lysis buffer containing 0.1% protease inhibitor (Sigma-Aldrich Co. LLC, USA) was added to the suspension and the mixture was sonicated. Subsequently, the protein in the samples was purified through four rounds of ITC. Then, the MW and purity of the proteins were confirmed using sodium dodecyl sulphate–polyacrylamide gel electrophoresis (SDS-PAGE). Furthermore, a UV–visible spectrophotometer (Agilent Technologies, CA, USA) was employed to measure the ELP concentration at 350 nm.

### Cell culture

Cells of the LLC cell line and the murine breast cancer cell line 4T1 were acquired from the American Type Culture Collection (ATCC) and grown in Roswell Park Memorial Institute (RPMI) medium (Hyclone, Invitrogen, Carlsbad, CA, USA) containing 10% fetal bovine serum (FBS; Gibco, Canada), 100 μg/ml penicillin, and 100 μg/ml of streptomycin (Sigma Aldrich). They were maintained at 37 °C in a humidified atmosphere with 5% CO_2_.

### Confocal microscopy

LLC and 4T1 cells (1 × 10^5^) were individually seeded onto a four-well chambered slide and grown until they reached 80% confluence. Subsequently, the cells were incubated with 0.5 μM fluorescein isothiocyanate** (**FITC)-labelled ELPs for 1 h each at 4 °C and 37 °C. After incubation, the cells were washed with PBS and fixed with 4% paraformaldehyde (Sigma Aldrich). Subsequently, their nuclei and plasma membranes were stained with DAPI and Wheat Germ Agglutinin, respectively, for 10 min, and the cells were immediately observed under a Zeiss LSM-510 META confocal microscope (Carl Zeiss, Germany).

### Cell binding assay

To assess the binding activities of the respective ELPs, 4T1 and LLC cells (1 × 10^6^ cells) were individually incubated with 0.5 μM fluorescein isothiocyanate** (**FITC)-labelled proteins for 1 h at 4 °C. Subsequently, the cells were washed three times with PBS to remove excess proteins and then suspended in 300 μL of PBS. The binding percentages were analyzed via flow cytometry (BD Bioscience, San Jose, CA, USA), with 10,000 events collected for each sample.

### Gel retardation assay

The miRNA-34a mimic (mature miRNA sequence: 5′-UGGCAGUGUCUUAGCUGGUUG U-3′), negative control (mature miRNA sequence: 5′-UUGUACUACACAAAAGUACUGA-3′), and carboxyfluorescein (FAM)-labeled FAM-anti-miRNA were procured from Bioneer, Daejeon, Republic of Korea. A gel retardation assay was performed to evaluate the complex formation between specific ELP polymers and miRNA. Negative control (NC) miRNA (10 μM) was mixed with 10–200 μM of ELPs to obtain samples with different miRNA/ELP molar ratios and a final volume of 40 μL. All samples were thoroughly vortexed and incubated at 25 °C for 30 min. Subsequently, the samples were subjected to electrophoresis on a 1% agarose gel and then stained with ethidium bromide. The formation of miRNA/ELP complexes was evident through the retarded migration of samples on the agarose gel. Designations for the complexes were based on their miRNA/ELP molar concentrations. For instance, in the designation 1:20, 1 and 20 correspond to 10 μM of miRNA and 200 μΜ of ELP (the highest concentration of ELP used), respectively.

### miRNA stability assay

For the miRNA stability test, 100 µM ELP and 5 μM of NC miRNA were mixed to form complexes. These complexes were then incubated with 1 μg RNase for 1 h at 37 °C. To disassemble the miRNA/ELP complexes, 7 µL of Heparin Sodium (JW Pharmaceutical, S. Korea) was added to the reaction mixture for 30 min. Subsequently, the samples were electrophoresed on a 1% agarose gel in tris–acetate-EDTA (TAE) buffer.

### TEM imaging

For TEM imaging, 20 µL miRNA-34a/ELP complexes solution was applied onto a carbon grid for 5 min. The excess solution was removed and the grid was air dried at room temperature. Subsequently, 20 µL 0.5% uranyl acetate solution was applied to the specimen for 5 min and the excess solution was removed. Lastly, the grid was air dried and characterized under Bio-TEM (Hitachi HT7700).

### miRNA/ELPs uptake assay

Flow cytometry was employed to quantitatively assess miRNA uptake. Briefly, LLC and 4T1 cells (1 × 10^6^ cells) were incubated with various fluorescein amidite (FAM)-labeled nanoformulations, including FAM-NC miRNA-Lipo2000, FAM-NC miRNA/Tat-E60, FAM-NC miRNA/A86, and FAM-NC miRNA/Tat-A86. Each formulation contained an equivalent amount of FAM—a chromogen-labeled miRNA (Bioneer, Daejeon, Republic of Korea). The cells were incubated with the nanoformulations for 1 h, then washed twice with PBS, centrifuged, and re-suspended in 500 μL of PBS. Finally, the mean fluorescence intensity (MFI) values, obtained using a flow cytometer, were tabulated and graphed for analysis.

### Lysosome staining

LLC and 4T1 cells were individually seeded onto a cover glass inside a six-well chambered dish filled with appropriate culture media. After 24 h, the cells were incubated with miRNA/ELP complexes (150 pmol of NC-miRNA labeled with FAM, Bioneer, Daejeon, Republic of Korea) for 1 h and 4 h at 37 °C. Subsequently, the cells were washed with PBS and incubated in pre-warmed culture medium containing LysoTracker Deep Red (Molecular Probes, Life Technologies Corporation, Eugene, OR, USA) for 1 h at 37 °C. Later, the cells were fixed with 4% paraformaldehyde, their nuclei were stained with DAPI, and observed under a confocal microscope.

### Cytotoxicity of miRNA/ELP complex

LLC and 4T1 cells (5 × 10^3^) were individually seeded into 96-well plates and allowed to grow for 24 h. Various concentrations (50, 100, 200, and 400 nM) of NC miRNA (MW: 13,323.78 Da) and miRNA-34a (MW: 14,019.55 Da) were encapsulated in ELPs at a molar ratio of 1:20 (10 μM miRNA and 200 μM of ELPs). Subsequently, the cells were treated with miRNA/ELP complexes for 6 h and further incubated with fresh complete media for 48 h. To assess the cytotoxicity effect, 10 μL of CCK-8 (Dojindo laboratories, Japan) solution was added to the wells, and the plate was further incubated at 37 °C for 1 h. Cells without miRNA/therapeutic treatment served as control. Finally, the absorbance of the cells was measured at 450 nm using a microplate reader.

### Cell cycle analysis

Exponentially growing LLC cells (5 × 10^5^) were plated onto a six-well plate. The following day, the cells were treated with miRNA/ELP (400 nM miRNA/8 μM ELPs) complexes at a ratio of 1:20 for 6 h. Subsequently, the culture medium was replaced with fresh complete media and incubated for 48 h. Next, aliquots of 1 × 10^6^ cells were fixed in 70% ethanol for 30 min at 4 °C and then centrifuged. Afterward, the cell pellets were stained with a solution containing propidium iodide (PI; 4 μg/ml) and RNase A (100 μg/ml) for 30 min. Finally, the cell cycle phases were analyzed using a fluorescence-activated cell sorter.

### Cell apoptosis analysis

To evaluate the anticancer efficacy of miR-34, apoptosis assays were conducted. Briefly, 5 × 10^5^ LLC and 4T1 cells were individually seeded into six-well plates and allowed to grow overnight. The cells were then treated with miRNA/ELPs (400 nM miRNA/8 μM ELPs) complexes at a ratio of 1:20 for 6 h. Subsequently, the culture media was replaced with fresh complete media. After 24 h of incubation, both floating and adherent cells were harvested, washed with PBS, and mixed with annexin V binding buffer (BD Biosciences, San Diego, CA, USA). Further, 2 μg mL^−1^ of PI and 5 μL of FITC annexin V reagent (BD Biosciences, San Diego, CA, USA) were added to stain necrotic and apoptotic cells. Annexin V- and PI-positive cells were quantified using flow cytometry.

### miRNA/ELPs inhibition in 3D model

Spheroid cultures of LLC cells were established in clear 96-well U-bottom plates with a cell-repellent surface. Each well was seeded with 10,000 LLC cells in 200 μL of culture medium, and spheroid formation was initiated through centrifugation at 1000 ×*g* for 10 min. Subsequently, the plates were incubated under standard cell culture conditions (37 °C, 5% CO_2_) in humidified incubators, allowing the cells to grow for 72 h to form multicellular tumor spheroids with an approximate diameter of 400 μm. To assess the inhibitory effect of miRNA-34a on tumor growth, LLC-tumor spheroids were subjected to treatments with either free ELPs or miRNA-34a/ELPs, each at miRNA concentrations of 5 μM and 10 μM equivalents. The diameter of the tumor spheroids in each well was determined using an inverted phase microscope (TS100-F, Nikon, Japan) for 10 days.

The cytotoxicity of the miRNA/ELP complexes in the spheroids was determined using a Live/Dead Viability/Cytotoxicity Kit (Biotium, Fremont, CA, USA). LLC spheroids with a 400 μm diameter were incubated with the complexes (miRNA-34a of 10 μM concentration). Following respective incubation periods of 3 and 6 days, the spheroids were gently rinsed with PBS and processed following the manufacturer’s protocol. Images were captured using a fluorescence microscope (TS100-F, Nikon, Japan).

### Animal experiment

All animal experiments were conducted following the guidelines of the Animal Care and Use Committee of Kyungpook National University (Permit Number: KNU 2022–0328). Experiments also followed the guidelines of the National Institute of Health (NIH) for the Care and Use of Laboratory Animals. Thirty female (six-week-old) C57BL/6 mice were housed in a specific pathogen-free environment. Tumors were induced through subcutaneous injections of LLC cells (1 × 10^6^ cells) into the right flank. Tumor cells were then left to grow for a week. When the tumor volume (V; mm^3^) reached approximately 100 mm^3^ (V = length × width^2^/2, measured using a Vernier caliper), the tumor-bearing mice were randomly divided into five groups (six mice per group) and were treated with the following: (i) PBS (0.1 mL); (ii) free miRNA-34a; (iii) miRNA-34a/Tat-E60; (iv) miRNA-34a/A86; and (iv) miRNA-34a/Tat-A86 at a dose of 6 mg/kg miRNA-34a. All formulations were administered via tail vein injection every other day for a total of five times. Tumor size and body weight were measured every 4th day. Finally, the mice were sacrificed 10 days after the final injection, and their tissues (tumor, heart, liver, spleen, lung, and kidney) were collected for further analysis.

### Terminal deoxynucleotidyl transferase (TdT)-mediated dUTP nick end labeling (TUNEL) assay

Tumor tissues from each group of mice were isolated and fixed with 4% paraformaldehyde overnight. Then, the tumor tissues were rapidly frozen and cryosections with a thickness of 8 μm were prepared from the frozen tumor tissues. Subsequently, the TUNEL assay (TUNEL Apo-Green Detection Kit, Biotool.com, Seoul, Korea), conjugated with fluorescein isothiocyanate (FITC), was performed following the manufacturer’s protocol to detect apoptosis. The number of TUNEL-positive cells was counted in 10 randomly selected microscopic fields.

### Hematoxylin and eosin (H&E) staining

After completing the therapeutic intervention, all major organs (including the liver, kidney, spleen, heart, and lungs) and tumor tissues were isolated from each treatment group and fixed with 4% paraformaldehyde. Paraffin-embedded blocks of each isolated tissue were prepared and sliced to a thickness of 3 μm. Then, H&E staining was performed to assess tissue morphology.

### Statistical analysis

Each experiment was independently repeated at least three times. Data are presented as the mean ± standard deviation (SD). Statistical significance was assessed using one-way analysis of variance (ANOVA) with the Graph Pad Prism 8 software, and statistical significance was set at P < 0.05.

## Results

### Synthesis and characterization of ELP nanoparticles for miRNA delivery

**Fig. 1:**
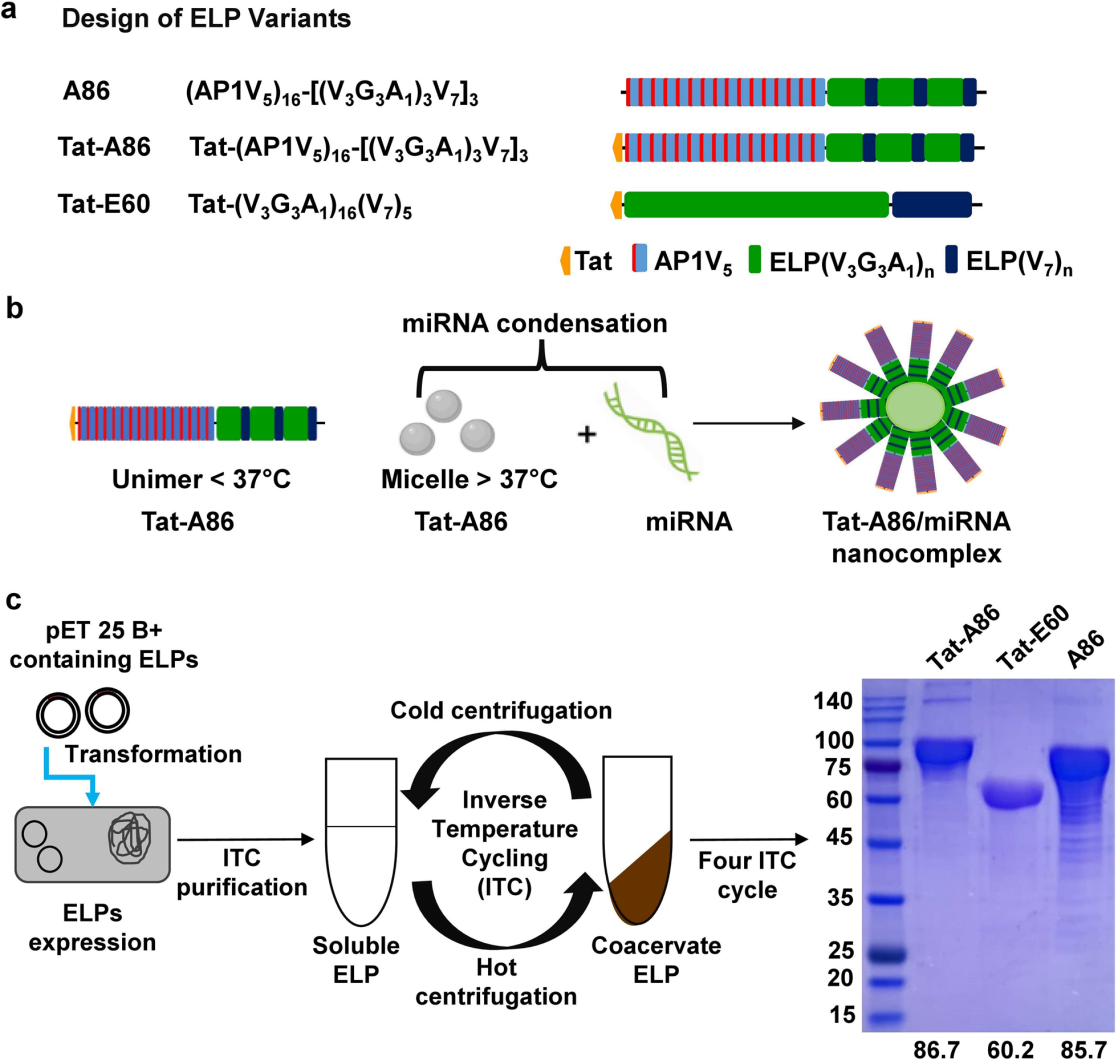
Design and expression of miRNA-binding ELPs. **a** Schematic representation of chimeric fusion ELPs encoding A86, Tat-E60, and Tat-A86, along with their corresponding amino acid sequences. **b** The carrier component of the ELP fusion protein is pivotal in mediating the interaction with miRNA. The positively charged amino acids in ELPs induce complexation with the negatively charged nucleic acids to form stable nano complexes. **c** pET 25b + vectors harboring Tat-E60, A86, and Tat-A86 genes were individually transformed into the *Escherichia coli* strain BL21 for ELP protein expression. Purification of all fusion ELPs involved triggering a phase transition and a repeated cycle of hot spin to aggregate protein, followed by cold spin to dissolve the ELP pellet, continued for four rounds to achieve homogeneity in fusion ELPs from cell lysate. The size and purity of the purified fusion proteins were confirmed through sodium dodecyl sulphate–polyacrylamide gel electrophoresis (SDS-PAGE), and Coomassie blue staining was employed for visualization. The size of each fusion ELP is indicated below the gel image

In pursuit of the development of a robust delivery system for miRNA-34a, built upon our innovative delivery platform utilizing multivalent ELP nanoparticles, this study reports on the synthesis, characterization, and application of Tat-A86 nanoparticles for the targeted delivery of miRNA-34a, with a particular focus on its utility in the context of replacement therapy for lung cancer. Additionally, this investigation underscores the comparative advantages of employing ELP nanoparticle-mediated methods over conventional naked miRNA delivery strategies. Notably, the versatility of the ELP-based polymer A86 enables the fine-tuning of drug delivery systems through genetic modifications and integration of functional peptides, such as the cell-penetrating peptide (CPP) Tat. In this study, Tat-A86 nanoparticles were synthesized by integrating Tat at the C-terminus of A86 (Fig. 1a). While A86 was composed of a 16-residue sequence designed as an interleukin-4 receptor-specific targeting ligand (IL4RP1, AP1) for the purpose of tumor-specific delivery, the inclusion of Tat ensured the efficient and direct delivery of miRNA genes to the cytosol. To validate the efficacy of Tat-A86, we established comparative groups, namely Tat-E60 (comprising only Tat and ELP) and A86 (comprising AP1 and ELP). The designed ELP consisted of pentapeptide repeats (Val-Pro-Gly-Xaa-Gly), where the Xaa residue represented valine (V), alanine (A), or glycine (G).

The positively charged arginine (R) residue in Tat (YGRKKRRQRRR) and AP-1 (RKRLDRN) of the different ELP variants facilitated DNA condensation, causing nanocomplex formation (Fig. 1b). Plasmids harbouring Tat-E60, A86, and Tat-A86 were individually transformed into competent *E. coli* BL21 cells. After high induction of protein expression with IPTG (1 mM), the ELPs protein were purified by exploiting the inverse phase cycling (ITC) method. Four cycles of repeated heating and cooling method of ITC followed by centrifugation, resulted in ELPs of high purities. Subsequently, the purity and MW of the proteins were determined through SDS-PAGE and Coomassie blue staining (Fig. 1c). The MW of Tat-E60, A86, and Tat-A86 was approximately 60.2 kDa, 85.7 kDa and 86.7 kDa respectively. The additional bands with MWs double the size of the expected ELP variants were likely a result of dimer formation, attributed to the presence of cysteine residues at the C-terminal.

### Analysis of the cell binding activities of ELP nanoparticles

**Fig. 2:**
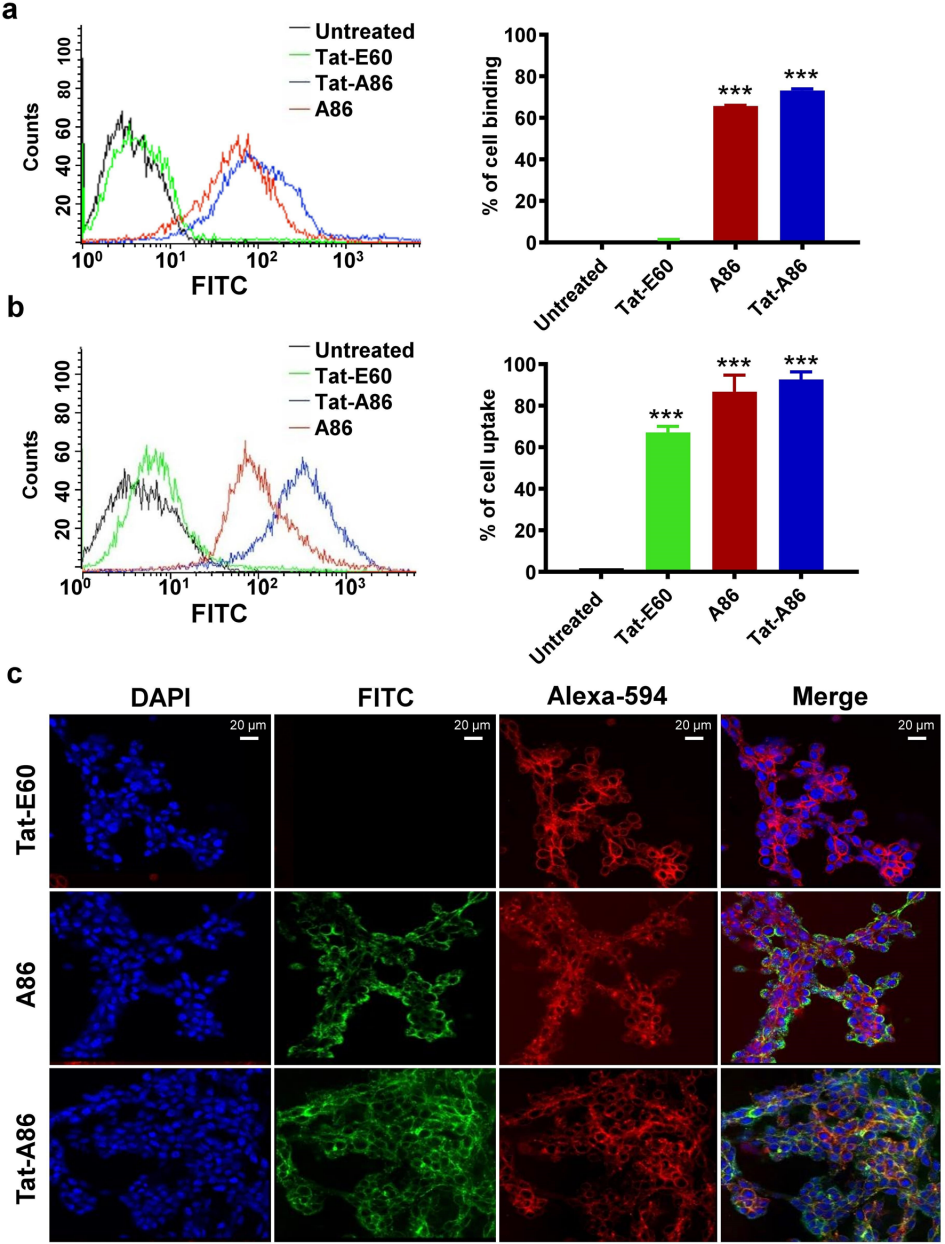
The binding of ELPs to LLC and 4T1 cells. LLC (**a**) and 4T1 (**b**) cells were incubated with fluorescein isothiocyanate** (**FITC)-labelled ELPs (0.5 µM) at 37 °C for 1 h. The percentage of cell-binding was analyzed through flow cytometry (*n* = 3). Graphical bars on the right represent the percentage of ELP-bound cells as the mean ± standard deviation (SD) of data obtained from three separate experiments performed in triplicate. ***P < 0.001 (one-way analysis of variance (ANOVA)) indicates significant differences for Tat-E60, A86 and Tat-A86 compared with untreated control. **c** LLC cells were treated with 0.5 µM of the respective fluorescein isothiocyanate** (**FITC)-labelled polypeptides at 37 °C for 1 h and observed under confocal laser microscopy. Cell membranes and nuclei were stained with Wheat Germ Agglutinin Alexa 594 and Hoechst, respectively. Representative confocal images from five independent experiments. Scale bar, 20 µm

The specificity and efficacy of the ELP nanoparticles were assessed before using them for targeted miRNA delivery to cancer cells. The cell binding activity of 0.5 μM of Tat-E60, A86, and Tat-A86 was evaluated on both LLC and 4T1 cells; the quantitative assessment was performed using flow cytometry. Tat-A86 demonstrated a higher binding percentage compared to the other ELPs in both LLC and 4T1 cells. The percentage of cell binding by Tat-E60, A86, and Tat-A86 in 4T1 cells were 1.05%, 65.7%, and 73.1%, respectively (Fig. 2a). In LLC cells, Tat-E60, A86, and Tat-A86 displayed 67.1%, 86.7%, and 92.6% binding activity, respectively (Fig. 2b). Notably, Tat-E60, which lacked AP-1, displayed considerably lower binding percentages in both cell lines. This disparity in binding efficiency underscores the importance of the AP-1 peptide in enhancing cell binding activity through IL-4R-mediated endocytosis.

To further validate the cellular uptake and internalization of the ELP nanoparticles, confocal microscopy was employed. LLC cells were incubated with FITC-labeled ELPs for 1 h each at 4 °C and 37 °C. At 4 °C, both Tat-A86 and A86 accumulated prominently on the cell surface, as evidenced by the distinct orange color, signifying the colocalization of the ELPs (green) and cell membranes (red) (Fig. 2c). Conversely, Tat-E60 displayed minimal cellular accumulation under the same conditions. At 37 °C, Tat-A86 showed more accumulation in the cytoplasm than in the membranes (Additional file 1: Figure S1). High-magnification images further confirmed the superior cytoplasmic accumulation of Tat-A86 compared to other ELPs. Overall, among the studied ELPs, Tat-A86 exhibited the best cell binding and uptake activities in LLC cells.

### Gel retardation and stability assay of ELP/miRNA complexes

**Fig. 3:**
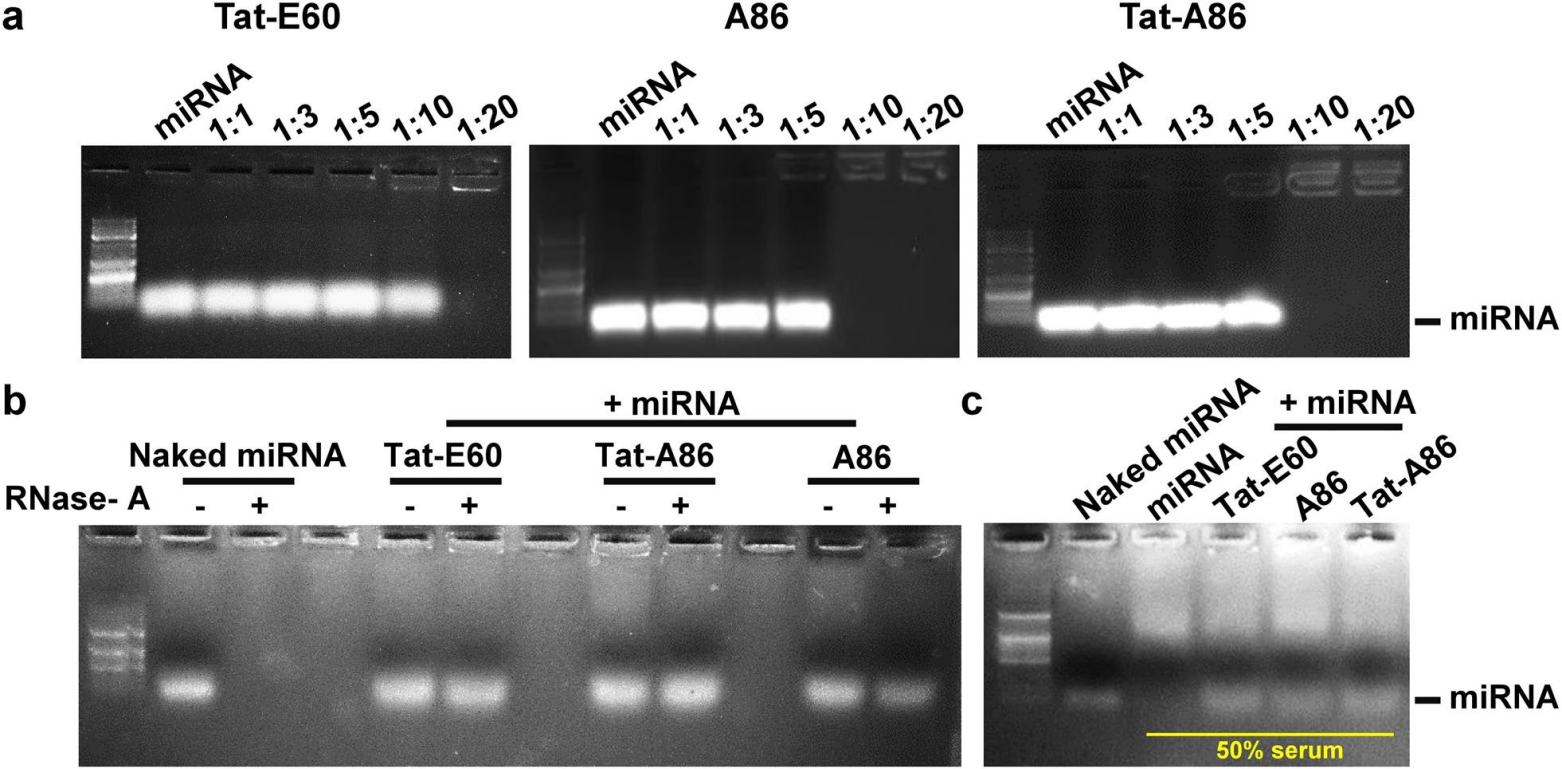
Gel retardation assay of miRNA/ELP complexes. **a** The complexation of Tat-E60, A86, and Tat-A86 with miRNA was examined at different miRNA/ELP ratios. Interactions between miRNA (10 μM) and ELPs (10–200 μM) were assessed using a gel retardation assay. The gels were visualized by staining with ethidium bromide. Lane 1: 1 kb DNA ladder, Lane 2: naked miRNA (22 bp), Lanes 3–7: miRNA/ELP complexes at different molar ratios. **b** The stability of encapsulated miRNA was evaluated by subjecting miRNA/ELP complexes to treatment with or without RNase A for 1 h. The samples were loaded alongside naked miRNA (Lane 2) and in the presence of RNase I (Lane 3). Lanes 5, 8, and 11 depict untreated Tat-E60/miRNA, Tat-A86/miRNA and A86/miRNA complexes, respectively. Lanes 6, 9, and 12 depict RNase A-treated Tat-E60/miRNA, Tat-A86/miRNA and A86/miRNA complexes respectively. Electrophoresis was performed on a 1% agarose gel in tris–acetate-EDTA (TAE) buffer, and gel visualization was achieved by staining with ethidium bromide. **c** A naked miRNA (Lane 2) or miRNA/ELPs complexes (Lane 3–5) were incubated with the 50% mouse serum for 24 h. Following incubation, the miRNA was liberated from the complexes utilizing heparin and subsequently subjected to analysis via agarose gel electrophoresis

The formation of miRNA/ELP complexes, a crucial aspect of successful gene delivery applications, was confirmed using a gel retardation assay. NC miRNA (10 μM) was mixed with varying concentrations (10, 30, 50, 100, and 200 μM) of ELPs to create miRNA/ELPs complexes with different molar ratios (1:1, 1:3, 1:5, 1:10, and 1:20). Increasing the miRNA/ELP ratios from 1:1 to 1:20 resulted in less migration of miRNA in the gel, indicating their entrapment in complexes (Fig. 3a). Both Tat-A86 and A86 efficiently encapsulated miRNA at a ratio of 1:10 (10 μM miRNA: 100 μM ELP), while Tat-E60 achieved complete complexation with miRNA at a ratio of 1:20. To enable a more comprehensive comparison and analysis in vitro, we implement a 1:20 ratio in subsequent experiments.

To further evaluate whether the formation of miRNA/ELPs complexes protected the miRNAs against RNase-mediated degradation, an RNase resistance assay was performed. Briefly, the complexes were incubated with or without RNase I for 1 h at 37 °C. As shown in Fig. 3b, naked miRNA was entirely digested by RNase I (Lane 3), while miRNA/Tat-E60, miRNA/Tat-A86, and miRNA/A86 (Lanes 6, 9, and 12, respectively) retained visible miRNA bands, indicating protection against RNase-mediated degradation. Among the studied ELPs, Tat-A86 (Lane 9) exhibited a notably high band intensity, indicating robust miRNA protection. When exposed to 50% FBS at 37 °C, naked miRNAs underwent rapid degradation (Fig. 3c). In contrast, miRNAs encapsulated by ELPs remained clearly visible even after an extended 24 h incubation period, highlighting the effective protection offered by ELPs in a biologically relevant environment.

### Characterization of miRNA/ELP complexes

**Fig. 4:**
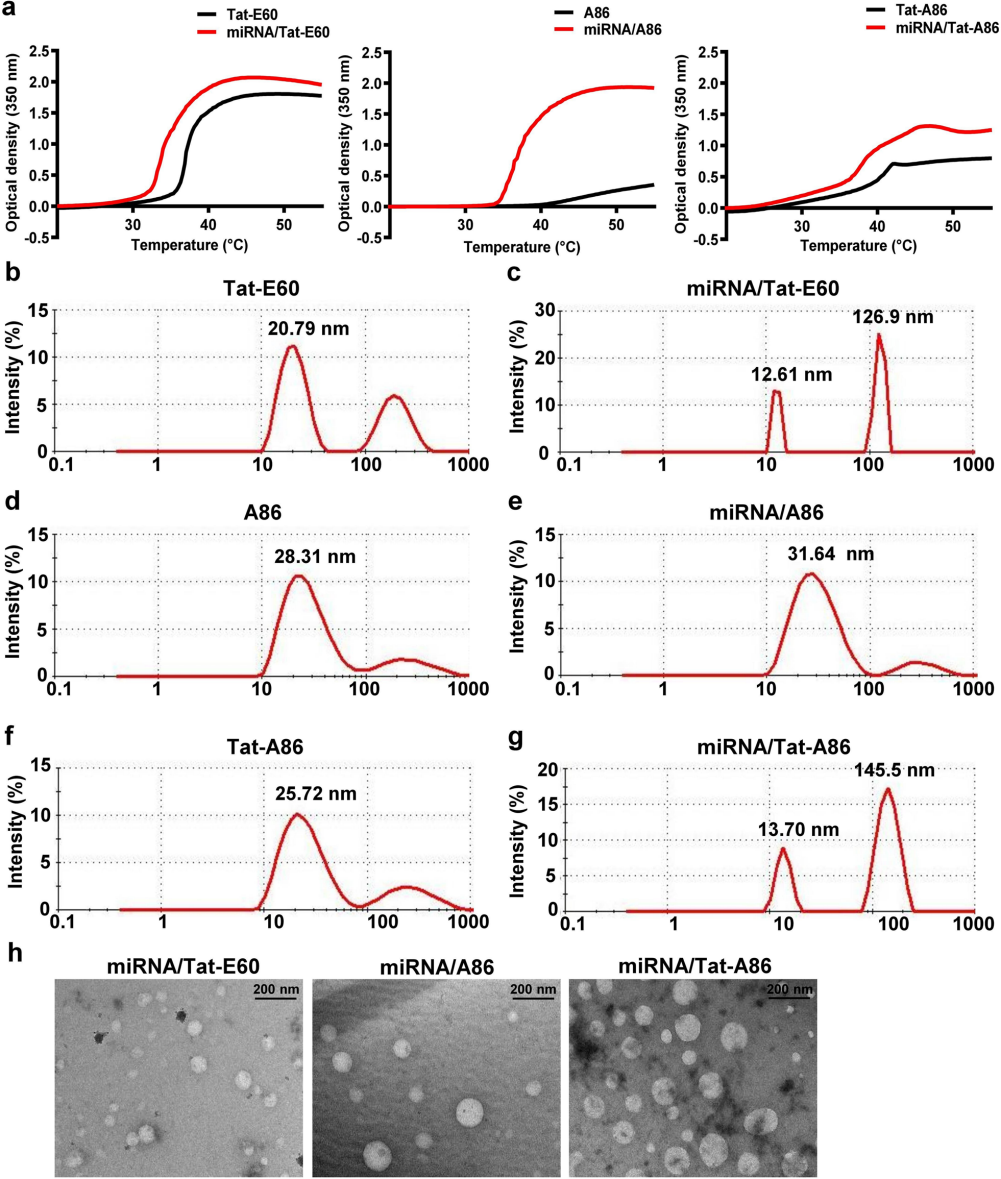
Physical characterization of miRNA/ELP complexes. **a** The turbidity profiles of the miRNA/ELP complexes (red) were assessed at 350 nm, with a temperature increase of 1 °C/min, and compared with those of naïve ELPs (black) at various temperatures. To validate the stable condensation of miRNA/ELP complexes, nanoparticle size was determined using dynamic light scattering (DLS) at 24 °C. The size parameters of ELPs alone and sizes following complex formation including Tat-E60 (**b**, **c**), A86 (**d**, **e**), and Tat-A86 (**f**, **g**) were evaluated. **h** TEM images of miRNA/Tat-E60, miRNA/A86 and miRNA/Tat-A86 nanocomplexes at room temperature. Scale bar = 200 nm

The transition temperature (Tt) of ELPs is a critical factor in their application for gene delivery. ELPs are biopolymers that exhibit reversible phase transitions, shifting from a soluble and disordered state to an insoluble and coacervate-like state in response to temperature changes. The Tt for each ELP and miRNA/ELP complex was measured at a rate of 1 °C per minute within a temperature range of 20 °C to 55 °C, as depicted in Fig. 4a. The Tt of Tat-E60, A38, and Tat-A86 were determined to be 37.2 °C, 46.47 °C, and 39.27 °C, respectively. Subsequent complexation with miRNA resulted in reduction of these temperatures to 34.47 °C, 37.37 °C, and 37.47 °C, respectively. The turbidity profile of Tat-E60 and Tat-A86 after complexation with miRNA revealed no significant changes in its Tt upon complex formation. Conversely, A86 demonstrated a significant decrease in Tt, with a reduction of 11.10 °C, signifying a substantial alteration in its transition behavior post-complex formation. This decline in Tt is ascribed to the electrostatic interactions between the positively charged residues on ELP and the negatively charged phosphate groups on miRNA, disrupting the hydrophobic interactions within the ELP polymer network. Consequently, the coacervate state of ELPs acts as a protective shield for encapsulated genetic material (e.g., DNA, RNA), playing a vital role in maintaining the stability and integrity of the genetic cargo in biological environments.

Parallel dynamic light scattering (DLS) measurements revealed that the particle size of miRNA complexes increased compared to that of the free peptides (Fig. 4b–g). The observed size and distribution characteristics of miRNA/ELP complexes are promising for in vivo systemic gene delivery applications. The particle size of both ELPs and miRNA/ELP complexes was measured at 24 °C. At 24 °C, Tat-E60 exhibited particles sizes of 20.79 nm and 203.1 nm (Fig. 4b). The presence of ELPs with two different particle sizes indicated the initiation of a unimer-to-aggregation phase transition. In contrast, A86 and Tat-A86 remained in a unimer state, exhibiting sizes of approximately 28.31 nm (Fig. 4d) and 25.72 nm (Fig. 4f), respectively. When condensed with miRNA, Tat-E60, A86, and Tat-A86 exhibited diameters of 126.9 nm (Fig. 4c), 31.64 nm (Fig. 4e), and 145.5 nm (Fig. 4g), respectively. The increase in particle sizes observed after miRNA encapsulation implies the successful condensation of miRNA by all three ELPs, leading to the formation of a nanoformulation. However, Tat-E60 and Tat-A86 exhibit larger particle size than A86, suggesting that Tat peptide might induce tighter packing or aggregation of the complexes, leading to larger observed sizes. Additionally, the structural characteristics of these nanoformulation using TEM clearly showed that Tat-E60, A86 and Tat-A86 retained their spherical micelle-like structures with average diameter sizes of 67.2 nm, 90.7 and 106.5 nm (Fig. 4h) respectively. The expected agglomeration within the dispersion is indicated by the noticeable size difference detected by DLS for Tat-E60 and Tat-A86 when compared to the dimensions observed in TEM images. The disparity in particle size between DLS and TEM is due to agglomeration, leading to larger hydrodynamic sizes in DLS, which includes the solvent shell, while TEM provides a direct measurement of particle size. For miRNA/A86 complexes, the average diameter sizes are typically larger in TEM analysis compared to DLS. It was speculated that A86 may undergo distinct dynamic structural changes or rearrangements in solution, potentially differing from Tat-containing complexes. Additionally, it was anticipated that A86 complexes might aggregate or form larger assemblies when dried onto the TEM grid, contributing to the observed larger sizes in TEM images. In contrast, DLS measures particle size in solution, where aggregation is less likely due to the presence of solvent molecules.

### Uptake of miRNA/ELP complexes by tumor cells

**Fig. 5:**
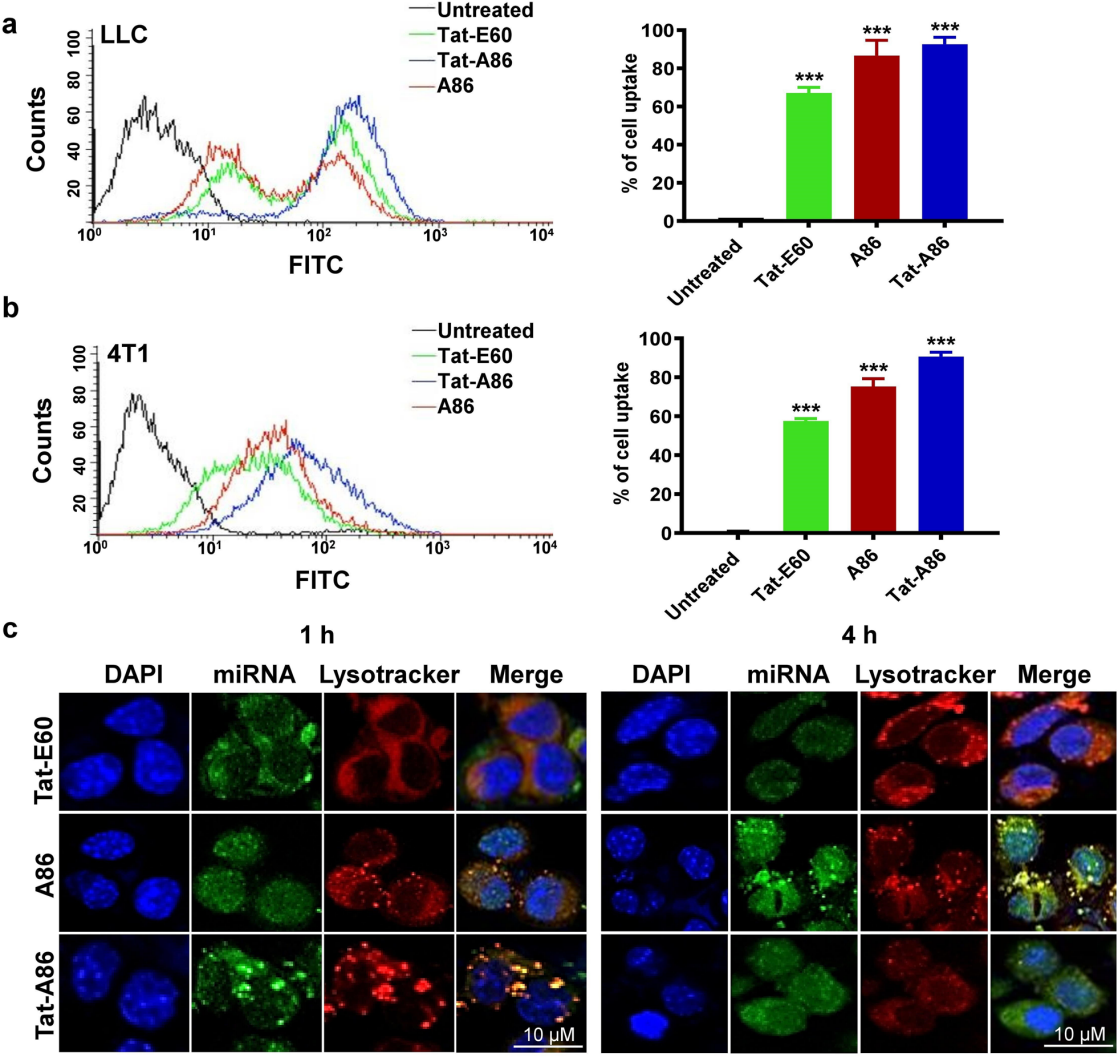
Cellular uptake and intracellular tracking of miRNA/ELP complexes. Uptake of FAM-labeled miRNA/ELP complexes by LLC (**a**) and 4T1 (**b**) cells was investigated. LLC and 4T1 cells were individually mixed with the complexes and incubated at 37 °C for 1 h. Subsequently, the miRNA uptake percentages were determined using flow cytometry (*n* = 3). Graphical bars on the right illustrate the mean ± SD of miRNA uptake percentages, derived from three independent experiments conducted in triplicate. ***P < 0.001, one-way analysis of variance (ANOVA) indicates significant differences for Tat-E60, A86 and Tat-A86 complexes compared with untreated control. **c** LLC cells were incubated with miRNA/ELP complexes containing 150 pmol of FAM-labeled miRNA for 1 h and 4 h. Lysosomal compartments were selectively labeled using LysoTracker Red DND-99, and the dynamic subcellular localization of miRNA was examined in real-time using a Zeiss confocal microscope. The yellow signal depicts the co-localization of miRNA with the lysosomal marker. Representative confocal microscopic images from five experiments (*n* = 5) are presented, with blue indicating nuclei stained with Hoechst, green representing miRNA/ELP complexes, and red corresponding to LysoTracker. Scale bar, 10 µm

Next, we investigated the cellular uptake and internalization of miRNA molecules upon their complexation with ELPs. To assess the uptake activity, labeled FAM-miRNAs (200 nM) were mixed with ELPs (4 µM) and individually incubated with LLC and 4T1 cells for 1 h at 37 °C. Subsequently, the uptake of the miRNA/ELP complexes was measured using flow cytometry. In LLC cells, miRNA/Tat-E60 demonstrated an uptake activity of 68.92%, while A86 exhibited a higher uptake activity at 86.73% (Fig. 5a). Notably, miRNA/Tat-A86 displayed the most significant uptake activity of 92.60%, higher than that of Tat-E60 and A86. Thus, ELPs, specifically A86 and Tat-A86, which contained IL-4R targeting peptides, markedly increased the cellular uptake of FAM-labeled miRNA. Similarly, in 4T1 cells, A86 and Tat-A86 revealed robust uptake activities of 75.31% and 90.65%, respectively. In contrast, miRNA/Tat-E60 complexes exhibited a 57.58% uptake activity (Fig. 5b). Overall, the exceptional effectiveness of Tat-A86 in promoting miRNA internalization within both LLC and 4T1 cells highlights its potential in miRNA-based therapies.

### Subcellular localization of miRNA/ELP complexes

The efficient cellular uptake of miRNA, its escape from subcellular compartments, and subsequent release into the target cytosol are pivotal considerations in the field of gene expression modulation, particularly in the domain of miRNA replacement therapy. Therefore, the efficacy of ELP carriers in facilitating miRNA translocation into the cellular milieu was assessed using confocal microscopy. FAM-labeled miRNA was used to directly visualize internalization patterns. The magnified images of cells treated with different miRNA/ELP complexes revealed both granular-shaped and diffuse signals. Granular signals, depicted in yellow, denoted the co-localization of miRNA (green signals) with lysosomal complexes (red signals), suggesting the sequestration of these miRNA molecules within lysosomes (Fig. 5c). Conversely, the diffuse miRNA signals scattered throughout the cytoplasm indicated active molecules that had escaped from lysosomal confinement. Interestingly, in LLC cells, Tat-A86 displayed the most pronounced co-localization with lysosomes, as evidenced by the conspicuous yellow spots resulting from the superimposition of green and red signals within the lysosomes after 1 h of incubation. This observation unequivocally indicated the internalization of miRNA/ELP complexes through an endocytic mechanism, with subsequent localization within the lysosomal compartments. However, after 4 h, the green signal was more pronounced in the cytoplasm, indicating the disruption of the lysosomal membrane and subsequent release of miRNA molecules into the cytoplasm. Contrarily, A86 displayed minimal localization within the lysosomes at 1 h but exhibited a substantial increase after 4 h, signifying a slower escape from the lysosomal compartment. In comparison, cells treated with miRNA/Tat-E60 complexes alone displayed faint miRNA signals primarily around the cell surface. This outcome may be attributed to the relatively weaker binding capacity of Tat-E60 or a possible loss of its cellular penetration ability after complexing with miRNA.

Similarly, in 4T1 cells, Tat-A86 exhibited the highest co-localization with lysosomes at 1 h (Additional file 2: Figure S2a), with subsequent accumulation in the cytoplasm after 4 h (Additional file 2: Figure S2b). Overall, these findings highlight the efficient delivery of Tat-A86 and its role in facilitating the gradual release of miRNA molecules, achieving the desired therapeutic effect.

### Cytotoxicity analysis

**Fig. 6:**
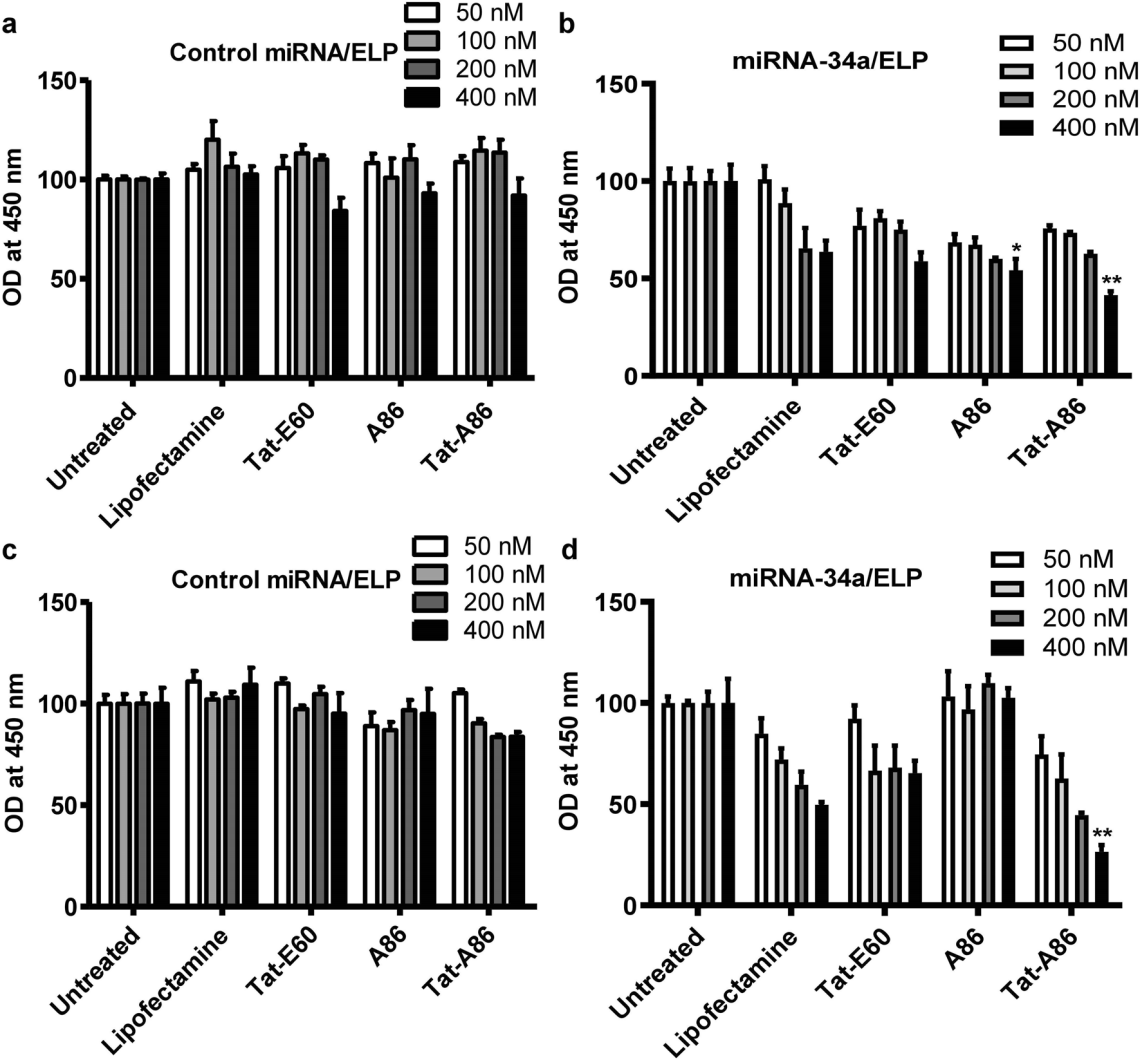
Cell viability assay. The impact of miRNA-34a on the growth of LLC (**a**, **b**) and 4T1 (**c**, **d**) cells was assessed following transfection with miRNA/ELPs. LLC or 4T1 cells (5 × 10^3^/well) in a 96-well plate were treated with negative control (NC) miRNA/ELPs and miRNA-34a/ELPs at concentrations ranging from 50 to 400 nM for 48 h. Cell proliferation was evaluated using a CCK assay. Specifically, 10 μL of CCK-8 solution was added to each well and incubated for 1 h at 37 ℃, and the change in absorbance was measured at a wavelength of 450 nm. One-way ANOVA was performed to assess the statistical significance of the differences among groups treated with miRNA/ELP complexes at different miRNA concentrations. *P < 0.05, NC miRNA/A86 vs. miRNA-34a/A86 (400 nM) delivery, and **P < 0.001, NC miRNA/Tat-A86 vs. miRNA-34a/Tat-A86-mediated miRNA (400 nM) delivery

miRNA-34a regulates several tumorigenesis-related genes, including Myc, CD44, MET, Bcl-2, and TP53 [45, 46], and its down-regulation or loss has been linked to the development of numerous types of cancer. Consequently, restoring miR-34a expression could potentially hinder tumor growth and progression, making it a promising candidate for cancer therapy [47, 48]. To evaluate the inhibitory effect of miRNA-34a, LLC and 4T1 cells were individually seeded into 96-well plates and treated with varying concentrations of miRNA (50, 100, 200, and 400 nM) at a 1:20 miRNA/ELP molar ratio. The cytotoxic effect of miRNA-34 was determined by comparing it to NC miRNA (Fig. 6a, c). After 48 h, CCK-8-based cell viability analysis revealed a dose-dependent reduction in cell viability in both LLC and 4T1 cells. Evidently, complexing tumor-suppressive miRNA-34a with Tat-A86 resulted.

in a higher cytotoxicity (41.45% cell viability) in LLC cells (Fig. 6b), surpassing the cytotoxicity observed for Tat-E60 (58.8% cell viability) and A86 (54.2% cell viability). Similarly, in 4T1 cells (Fig. 6d), Tat-A86 complexed with miRNA-34a exhibited the highest inhibition (26.45% cell viability), as opposed to Tat-E60 (65.36% cell viability) or A86 (102% cell viability). Overall, miRNA/Tat-A86 complexes demonstrated a more potent inhibitory effect compared to miRNA/Tat-E60 or miRNA/A86 complexes. Treatment of LLC cells with free ELPs did not result in any inhibitory effects (Additional file 3: Figure S3). Furthermore, NC miRNA complexed with ELPs had little to no impact on cell viability in both cell lines (Fig. 6a, c). Thus, this study clearly demonstrates the efficient delivery of miRNA by Tat-A86.

### Effect of miRNA‑34a on LLC cell cycle distribution and apoptosis

**Fig. 7:**
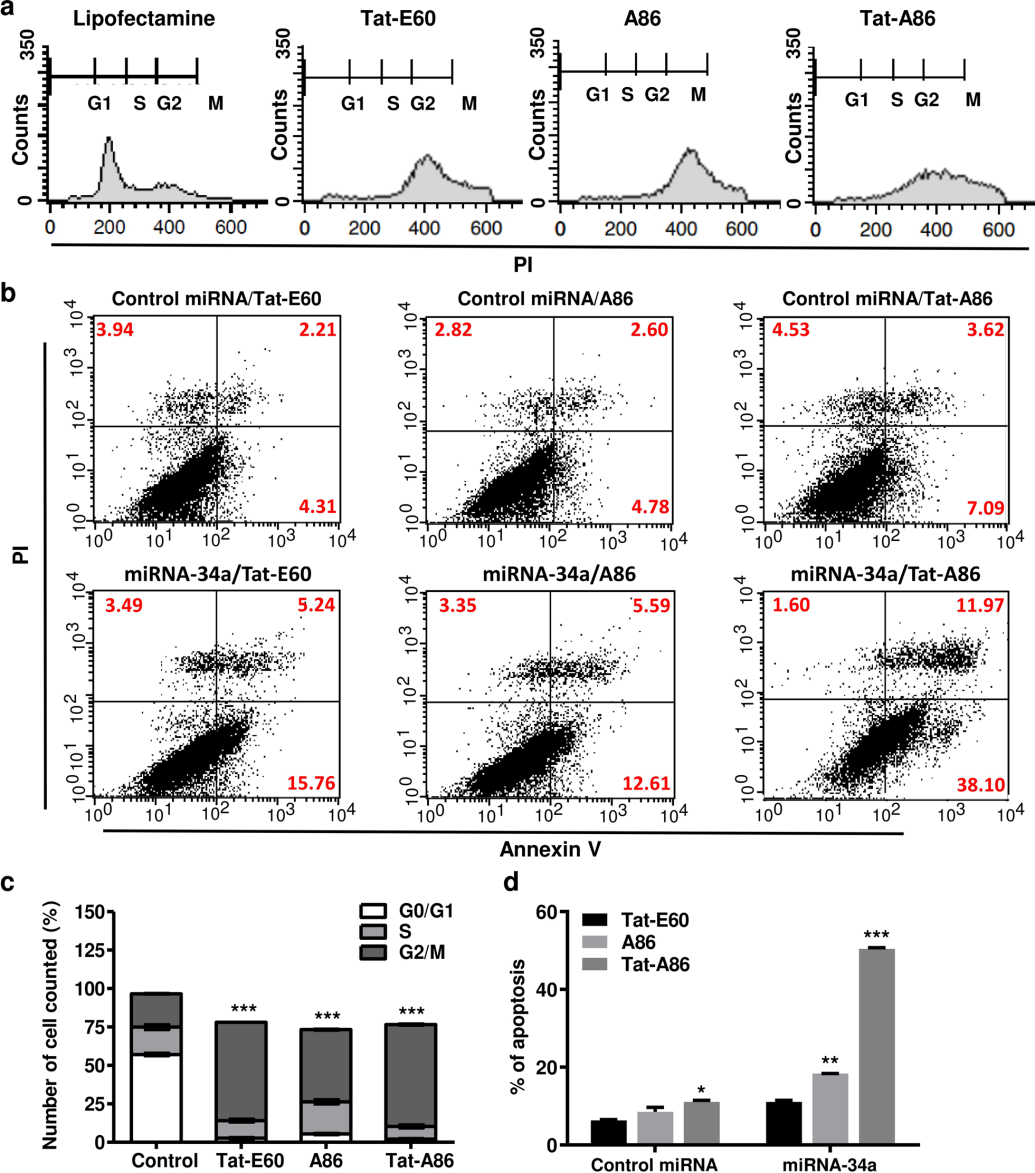
miR-34a/ELPs induce G2/M phase arrest and cellular apoptosis. **a** LLC cells were transfected with miRNA-34a/ ELP, and the cell cycle distribution was analyzed 48 h post-transfection using flow cytometry after propidium iodide (PI) staining. **b** Flow cytometry analysis, followed by annexin V–PI staining, revealed the apoptosis percentages in LLC cells treated with different miRNA-34a/ELPs. Representative images from the flow cytometry analysis are shown. **c** The percentages of cell populations in the G0/G1, S, and G2/M phases were analyzed relative to the total phases. Most miRNA-34a/ELPs induced cell cycle arrest at the G2/M phase in LLC cells. Data are presented as the mean ± SD from at least three independent experiments. ***P < 0.001 indicates statistically significant difference in the miR-34a/Tat-E60, miR-34a/A86 and miR-34a/Tat-A86 -treated group compared to the control group. **d** The percentage of apoptotic cells increased significantly in miRNA-34a/Tat-A86-treated LLC cells compared to the control. Data are presented as the mean ± SD for relative apoptosis normalized to control cells for three independent experiments. Columns represent the mean of three separate experiments, while bars represent the SD. **P < 0.01, ***P < 0.001 as determined by ANOVA test, miRNA-34a/Tat-E60 vs. miRNA/miRNA-34a/A86, and miRNA-34a/Tat-E60 vs. miRNA-34a/Tat-A86

miRNA-34 family members, being direct p53 targets, induce apoptosis and cell-cycle arrest [49]. To gain further insights into the mechanisms underlying the heightened cell cycle distribution and apoptosis rates facilitated by miRNA-34a/ELPs, their effects on tumor cells were investigated. Specifically, the impact of miR-34a upregulation on LLC cell cycle was examined, revealing G1 cell cycle arrest (Fig. 7a). Transfecting LLC cells with 400 nM miR-34a using lipofectamine resulted in a G1-G0 arrest in 57.89% of the cell population (Fig. 7c). Remarkably, transfection with miRNA-34a/ELPs led to the majority of LLC cells arresting in the G2 phase. Complexing miRNA-34a with Tat-E60, A86, and Tat-A86 at the same 400 nM miRNA concentration induced M/G2 arrest in 74.55%, 67.87%, and 75.24% of the cell population, respectively. No significant differences were observed in the relative proportion of cells in the G2 phase in the miRNA-34a/ELP-treated groups at 48 h post-transfection.

Additionally, analysis of the cell-killing activity of miRNA-34a/ELPs in LLC cells through annexin V assays (Fig. 7b) revealed that miRNA-34a/Tat-A86 had significantly higher cell-killing activity (~ 50.40%) compared to the untreated and miRNA-34a/ELP controls (Fig. 7d). Furthermore, treatment with miRNA-34a/Tat-E60 and miRNA-34a/A86 induced at least 11.09% and 18.35% apoptosis, respectively, indicating a moderate impact. Overall, miRNA-34a/Tat-A86 notably increased the cell populations in the G2/M-phase, reduced the cell populations in the S-phase, and significantly elevated the total cell death rate due to apoptosis.

Furthermore, the impact of miRNA-34a/ELPs on the tumorigenic potential of 4T1 cells was assessed via a colony formation assay (Additional file 4: Figure S4a). Treatment with miRNA-34a/Tat-A86 reduced colony formation by ~ 48.8% compared to the PBS-treated control group (Additional file 4: Figure S4b, S4c). As expected, NC miRNA/ELPs had no direct cell-killing effect on 4T1 cells. Collectively, the results of the anticancer activity assays affirm the promising activity of miRNA-34a, which is closely linked to its effective delivery via Tat-A86 nanoparticles.

### Penetration and anti-tumor effects of miRNA-34a/ELPs in the LLC spheroids model

Given the lack of knowledge regarding the optimal systemically administered dose of miRNA-34a and its success rate in reaching the tumor tissue, the effects induced by miRNA-34a in an LLC-3D spheroid model were initially examined. For this purpose, first, LLC spheroids with varying cell numbers (5,000, 10,000, and 15,000 cells) were prepared, and their growth was monitored at different time points (Additional file 5: Figure S5a, S5b). The spheroid diameter in all groups exhibited a gradual increase after an initial slight decrease (Additional file 5: Figure S5a). Cell viability within the spheroids was examined using a Live/Dead Cell Assay Kit. The Live/Dead Cell Assay Kit facilitated the discrimination of live cells from dead ones by simultaneously staining live cells with green-fluorescent calcein-AM (indicating intracellular esterase activity) and dead cells with red-fluorescent ethidium homodimer-1 (which binds to intracellular nucleic acids). The results revealed that cells within 10-day-old spheroids retained their viability in the case of spheroids containing 5,000 or 10,000 cells per micro-well (Additional file 6: Figure S6a-c). Conversely, spheroids with 15,000 cells per micro-well displayed a notable decrease in cell viability. Therefore, spheroids generated from 10,000 cells per micro-well were selected for subsequent evaluations, considering their more favorable characteristics.

The delivery of FAM-labeled miRNA facilitated by ELPs was also assessed, specifically examining its penetration within the LLC spheroid at 37 °C. This was subsequently confirmed via confocal microscopy, as depicted in Figure S7 (Additional file 7). It was illustrated that the fluorescence was primarily localized along the periphery of the spheroid when treated with the FAM-labelled miRNA complex with ELPs. A significantly higher fluorescence intensity was detected in multicellular tumor spheroids treated with Tat-A86, indicating the enhanced penetration capability of miRNA/Tat-A86, as anticipated. Furthermore, both Tat-E60 and A86 exhibited limited penetration, resulting in lower FAM intensity within the spheroid. These results highlight the superior tumor tissue penetration ability of Tat-A86 (A86 modified with Tat) compared to regular Tat-E60 or A86.

**Fig. 8:**
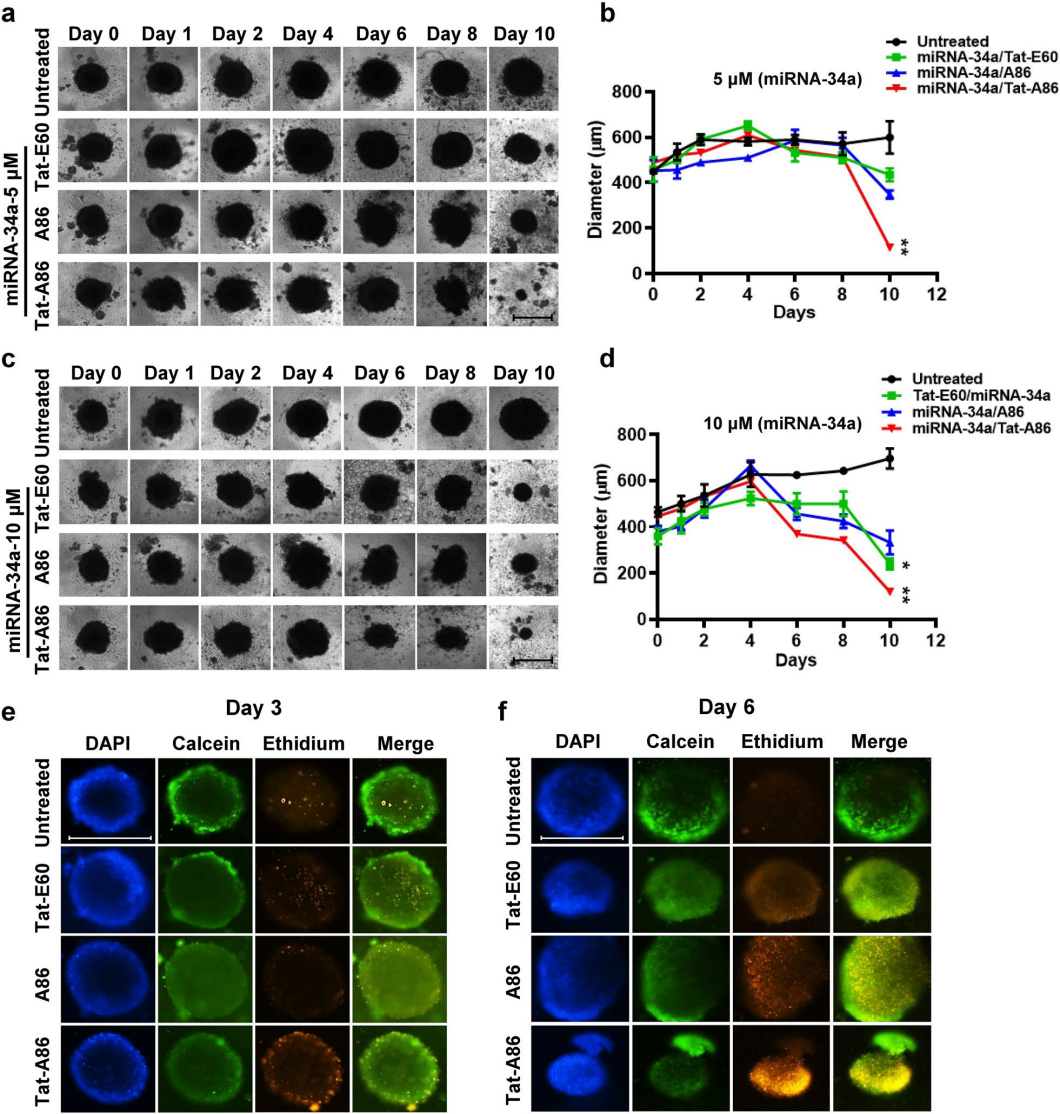
Growth inhibition assay using the LLC spheroids. **a**–**d** LLC spheroids were treated with different miRNA/ELP complexes, featuring varying miRNA-34a concentrations (5 or 10 μM) encapsulated within different ELPs. The time-dependent increase in the diameter of LLC spheroids was monitored for 10 days using bright-field imaging. Representative images of spheroid treated with miRNA/ELP complexes are presented. LLC spheroids cultured in Roswell Park Memorial Institute (RPMI) medium served as a control. Scale bar, 500 μm. Growth curve of LLC spheroids after various treatments. **P < 0.01, Tat-A86/miRNA-34a treated vs. control group from day 6 onwards. **e**, **f** Cell viability assay of the differently sized spheroids (day 3 and day 6) using live/dead staining with ethidium homodimer-1 and calcein AM. Viable cells are depicted in green, while nonviable cells are shown in red. Scale bar, 500 μm

Next, the cytotoxicity of miRNA-34a/ELP complexes at different miRNA-34a concentrations (5 μM and 10 μM) was assessed by examining their capacity to suppress the growth of tumor spheroids. Particularly, tumor spheroids incubated in complete media displayed consistent growth, with the spheroid volume increasing over the 10-day incubation period (Fig. 8a, b). At a miRNA-34a concentration of 5 μM, the miRNA-34a/Tat-A86 exhibited a gradual impact on tumor inhibition with reduction in spheroid size on day 10. Conversely, exposure to miRNA-34a/Tat-A86 at a concentration equivalent to that of miRNA-34a (10 μM) resulted in significant inhibition of tumor spheroid growth, manifested by alterations in shape from day 6 onward (Fig. 8c, d), with a noteworthy decrease in spheroid size on day 10 post-treatment. The results unveiled a dose-dependent response of tumor cells to miRNA-34a, with higher concentrations significantly reducing cell viability compared to lower concentrations. Interestingly, groups treated with miRNA-34a/Tat-E60 or miRNA-34a/A86 displayed the slowest tumor spheroid growth rates in a dose-dependent manner, leading to a subsequent reduction in tumor size on day 10 post-treatment.

Furthermore, when using ELPs alone at concentrations of 100 and 200 μM for encapsulating miRNA-34a (5 μM and 10 μM), minimal to negligible effects on the growth of LLC spheroids were observed (Additional file 8: Figure S8a–c). This outcome clearly indicates that the observed inhibition in spheroid growth is specifically attributable to the facilitation of miRNA-34a delivery by ELPs. The superior tumor inhibition achieved through miRNA-34a/Tat-A86 is attributed to the exceptional tumor-penetrating ability of Tat-A86.

In congruence with observed morphological alterations, microscopic imaging of live (Calcein AM) and dead (Ethidium homodimer) cell populations within LLC spheroid cultures subjected to specified concentrations of 10 μM miRNA-34a substantiates the efficacy of miRNA-34a/Tat-A86-mediated tumor inhibition. Remarkably, within the Tat-A86 treated cohort, discernible apoptosis of tumor cells manifested at the periphery of the spheroids after 3 days of treatment (Fig. 8e). This phenomenon was accompanied by a reduction in live cell staining coupled with a statistically significant elevation in dead cell staining by day 6 (Fig. 8f). Conversely, neither miRNA-34a/A86 nor miRNA-34a/Tat-E60 treated LLC spheroids exhibited an augmentation in dead staining at 3 days post-treatment, although discernible changes were observed by day 6. Untreated cells-maintained viability at both day 3 and day 6, as indicated by sustained intense green fluorescence.

### In vivo therapeutic effect

**Fig. 9:**
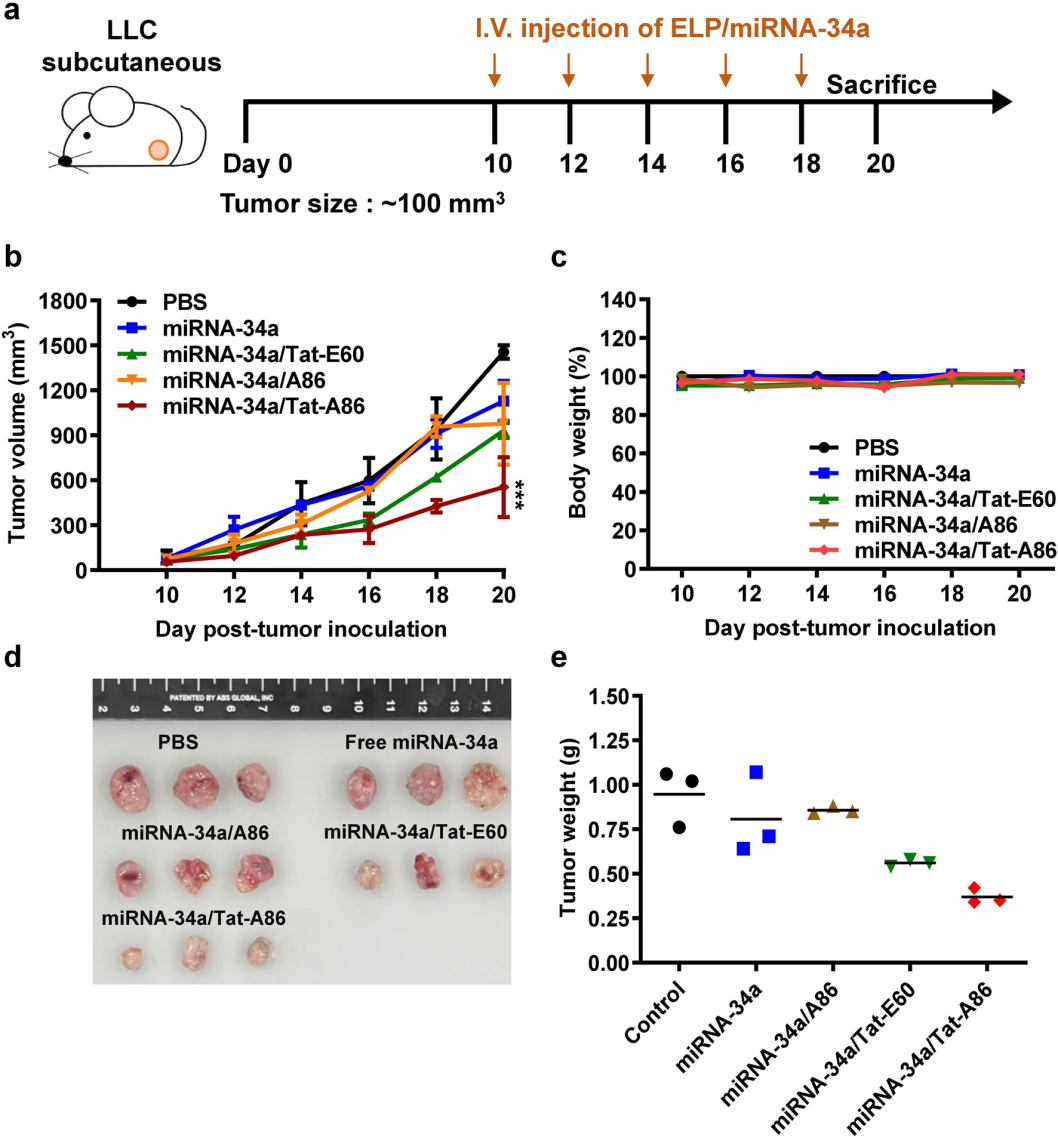
The antitumor effect of miRNA-34a/ELPs in vivo. **a** LLC allograft mice were randomly divided into five groups and administered intravenous injections of either free miRNA-34a (6 mg/kg) or miRNA-34a/ELPs (6 mg/kg on days 1, 3, 6, 8, and 10). Tumor volumes (mm^3^) were monitored every after 3 days for 20 days. **b** Tumor volumes are presented as mean ± SD (*n* = 5 per group). **c** The average body weight of each group is expressed as the mean ± SD (*n* = 5 per group). **d** Tumors in each group, including the control (PBS), free miRNA-34a, Tat-E60, A86, and Tat-A86 groups, were excised and photographed after 20 days. **e** Average tumor weight in each group at the end of the experiments. Columns represent the mean for three replicate determinations, while bars indicate SD. ***P < 0.001 indicates the statistically significant difference in the miRNA-34a/Tat-A86-treated group compared to the control PBS group

To assess whether the systemic delivery of miRNA-34a or miRNA-34a/ELPs could impede lung tumor growth in LLC-bearing C57BL/6 mice, miRNA-34a or miRNA-34a/ELP formulations were administered to subcutaneous tumors via intravenous tail vein injections. The treatment commenced 10 days after tumor implantation, corresponding to a tumor size of approximately ~ 100 mm^3^. Each dose contained 10 μM of formulated miRNA-34a, equivalent to (6 mg/kg) per mouse with an average weight of 20 g, and was administered every other day for a total of five doses (Fig. 9a). Systemic intravenous treatment with miRNA-34a/Tat-A86 nanoparticles effectively inhibited tumor growth (~ 38%) compared to PBS (Fig. 9b). In contrast, treatment with control miRNA-34a had a considerably weaker effect on tumor growth by 77.5%. Likewise, the other control groups, Tat-E60 (64%) and A86 (67%), demonstrated relatively modest reductions in tumor growth compared to Tat-A86. These findings highlight that free miRNA-34a had a limited impact on the growth of LLC tumors. Conversely, miRNA-34a delivery using Tat-A86 nanoparticles resulted in substantial antitumor activity. After completing the therapeutic regimen, the excised tumors were photographed and weighed (Fig. 9d). The mean tumor weight in the miRNA-34a/Tat-A86 (0.37 g) group was significantly lower than that in the miRNA-34a/Tat-E60 (0.56 g), miRNA-34a/A86 (0.85 g), and untreated (~ 0.94 g) groups (Fig. 9e). These findings confirm the strong antitumor effect of miRNA-34a/Tat-A86 nanoparticles in LLC. Furthermore, miRNA-34a/Tat-A86 had no apparent signs of gross toxicity, as evidenced by the unchanged body weight during the treatment (Fig. 9c).

**Fig. 10:**
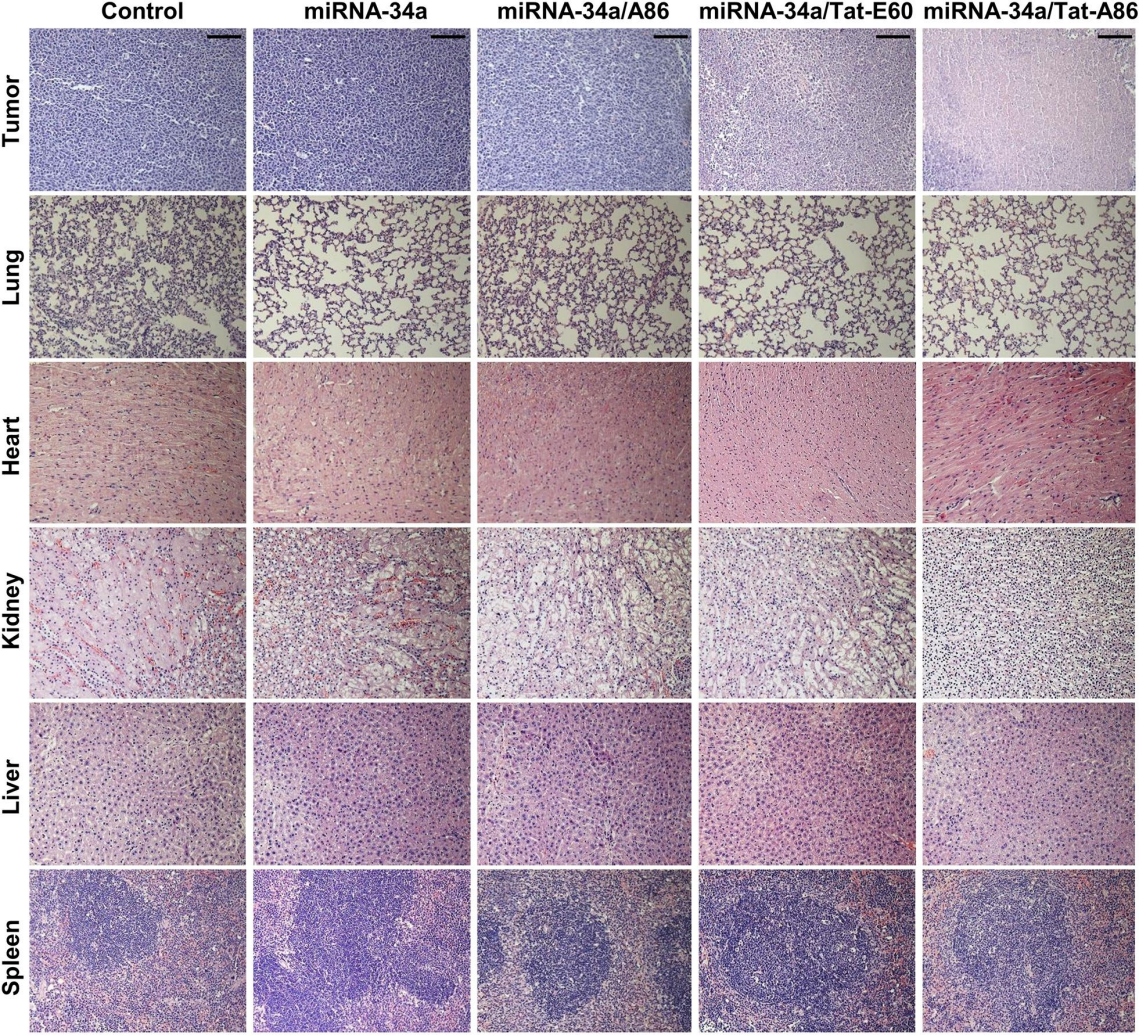
Ex vivo histological analyses based on hematoxylin and eosin (H&E) staining. Following the treatment regimen, tumor sections were excised from the LCC tumor-bearing mice. Nuclei are stained in blue, while the extracellular matrix and cytoplasm are stained in red using H&E staining. Scale bar, 50 μm

**Fig. 11:**
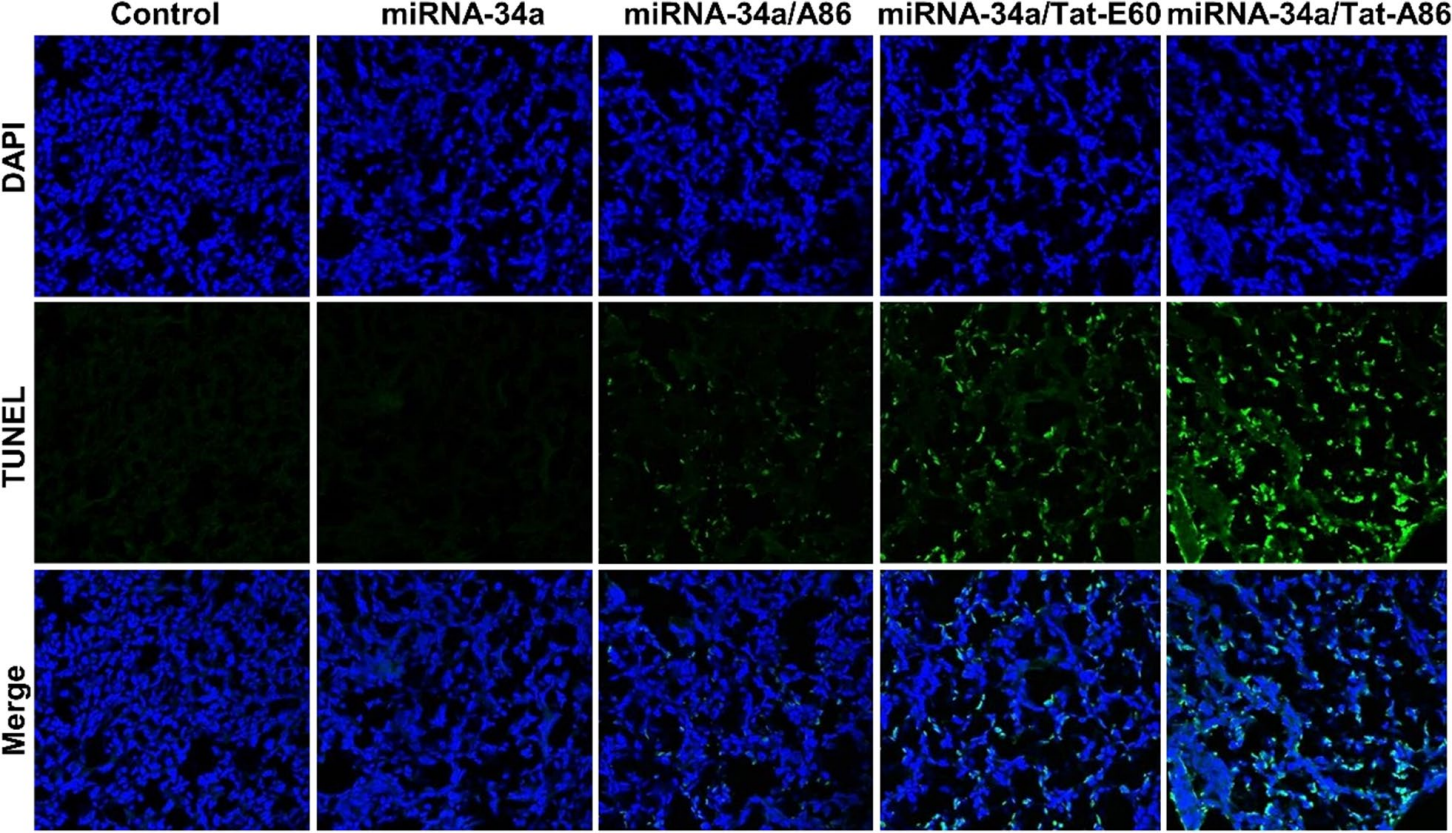
Analysis of apoptosis based on terminal deoxynucleotidyl transferase (TdT)-mediated dUTP nick end labeling (TUNEL) assay. Apoptosis in tumor tissues across various groups (*n* = 5) was assessed through TUNEL staining. Apoptotic cells are represented by green spots, while intact DNA is marked in blue stain using DAPI. Scale bar, 50 µm

Moreover, H&E staining of major organs revealed no obvious tissue damage in the treatment groups compared to the control (PBS-treated) group (Fig. 10). Due to the low dosage of miRNA-34a (6 mg/kg) employed in this study, no decrease in body weight or occurrence of tissue injury was detected throughout the therapy. However, analysis of the H&E tumor sections suggested considerably enhanced cell death in the miRNA-34a/Tat-A86-treated group compared to the control group. TUNEL staining of the tumor tissues validated the apoptotic activity of the miRNA-34a/ELP formulations (Fig. 11). The miRNA-34a/Tat-A86-treated group showed higher apoptotic activity compared to the PBS-treated group. TUNEL-positive cells were observed in both miRNA-34a/Tat-E60 and miRNA-34a/A86 groups but to a lesser extent than that in the Tat-A86 group.

## Discussion

We pioneered a robust miRNA delivery platform, addressing the intricate challenges related to miRNA replacement therapy, including the imperative for tissue-specific delivery and concerns regarding safety and potential off-target effects. Considering the critical impact of the MW and size of nanoparticles on the efficacy and safety of miRNA delivery systems, we employed a recombinant ELP-based multivalent targeting approach. In our preliminary investigations, we highlighted the enhanced binding activity of the high molecular weight A86 nanoparticles (38–40 nm in diameter) transitioning to a micelle-like structure. Furthermore, in vivo assessments across diverse mouse models reaffirmed the promising potential of A86 in tumor localization and retention. Acknowledging the demand for precise miRNA delivery to specific cellular locations, we sought to enhance the therapeutic performance of A86 by fusing it with Tat, a short CPP derived from human immunodeficiency virus. The resulting fusion, Tat-A86, ensured successful miRNA transport to intracellular locations, enabling the modulation of miRNA expression and activity, and paving the way for potential advancements in cancer treatment.

In this study, we investigated the potential of our novel multidomain ELP nanoparticle, Tat-A86, as a carrier for miRNA-34a delivery. Through recombinant DNA engineering, Tat-A86 and other ELP variants were precisely designed for optimal gene delivery and easy purification (using the *E. coli* bacterial system). The phase transition characteristic of ELPs enabled their isolation using the simple, inexpensive, and non-chromatographic batch method—ITC. Additionally, the ELPs were engineered to possess a Tt matching physiological conditions, enabling controlled release, genetic material protection, enhanced cellular uptake, and targeted gene delivery to specific tissues or cells.

Prior to miRNA delivery, we assessed the capacity of Tat-A86 to target specific tumor cells. In vitro flow cytometry analysis revealed that both Tat-A86 and A86 exhibited significantly enhanced binding to LLC and 4T1 murine cancer cells compared to Tat-E60 (Fig. 2a, b). Consistent with these findings, confocal imaging clearly revealed the substantial cellular accumulation and uptake of Tat-A86 and A86, while Tat-E60 exhibited minimal accumulation. This observation emphasized that the improvements were primarily attributed to the presence of AP-1 rather than nonspecific penetration by Tat (Fig. 2c and additional file 1: Figure S1).

Next, we investigated the condensation of miRNA with Tat-E60, A86, and Tat-A86, revealing successful encapsulation at different molar ratios (Fig. 3a). The interaction was facilitated by binding of the positively charged amino acids in Tat to the negatively charged miRNA. Notably, condensation at a lower molar ratio, particularly in the case of Tat-A86, may be attributed to the higher positive charge conferred by the arginine residue of AP1. Importantly, the encapsulation of miRNA by the ELPs effectively shielded it from RNase A degradation (Fig. 3b), with Tat-A86 exhibiting superior miRNA protection when compared to the other ELPs. The substantial number of positively charged arginine residues in Tat-A86 strengthened its miRNA binding, thereby enhancing its miRNA encapsulation capacity and protection. In contrast, Tat-E60 exhibited nucleic acid condensation at a higher molar ratio owing to the absence of AP1. This suggests that ELP does not directly interact with nucleic acids but primarily imparts thermal sensitivity and stability. Thus, the increased number of arginine residues in Tat-A86 contributes to the overall stability of the nanocomplex.

The imperative retention of ELP phase transition characteristics post-condensation with genetic materials is essential for effectively facilitating targeted gene delivery. The conspicuous decrease in the Tt indicated an intensified hydrophobic effect during the complex formation process. This reduction in Tt is attributed to the interaction of positively charged ELPs with the negatively charged miRNA, leading to the establishment of a highly hydrophobic state (Fig. 4a) [50]. Furthermore, the positive charges on ELPs play a crucial role in neutralizing the negative charges on miRNA, thereby contributing to the sustained hydrophobicity and, consequently, the observed depression in Tt. This intricate interplay between ELPs and miRNA highlights their potential in forming stable nanocomplexes for gene delivery applications. This observation was further substantiated by conclusive evidence from concurrent DLS and TEM analysis. The DLS results delineated the enlargement in particle sizes after miRNA condensation, offering additional confirmation of the successful formation of stable nanocomplexes (Fig. 4b–g). These cumulative insights underscore the significance of comprehending and preserving ELP phase transition properties within the gene delivery paradigm, establishing a robust foundation for efficacious therapeutic interventions at anatomically precise sites.

The cellular uptake of miRNA/ELP complexes, their subsequent release from subcellular compartments, and their eventual delivery into the target cytosol are pivotal factors in the field of gene expression regulation. Therefore, analyzing the interaction of ELP variants with cellular membranes and their subsequent cellular entry is significant, especially in the context of miRNA replacement therapy. Thus, we assessed the effectiveness of ELPs in facilitating the translocation of miRNA into the cellular milieu using confocal microscopy. To directly visualize internalization patterns, we employed FAM-labeled miRNA. Colocalization of the nanocomplex was confirmed through the merged green fluorescence between the red fluorescence (LysoTracker) of the lysosome (Fig. 5c). The colocalized miRNA/ELP complex was observed to be distributed around the nucleus in the form of particles in the cytoplasm. These forms represent a lysosome containing nanocomplex. In contrast, the spread of green fluorescence might indicate that miRNAs dissociated from nanocomplexes after escaping from lysosomes. Interestingly, the miRNA/Tat-A86 complex exhibited the highest localization in lysosome within 1 h, followed by dissociation after 4 h. Since miRNA/Tat-A86 formulations can be internalized through endocytosis, they efficiently approach the cell membrane and, crucially, evade lysosomal degradation. Consequently, the complex efficiently transports miRNA into the cytoplasm. This shows that Tat-A86, with its multiple copies of AP1 ligand and Tat peptide, can easily target tumor cells. This observation supports the hypothesis that Tat-A86 is advantageous for targeted miRNA delivery. Therefore, this formulation possesses favourable properties for improved miRNA delivery and cellular uptake, potentially contributing to its anticancer properties.

The restoration of miRNA-34a activity has been established as an effective strategy to impede cancer cell proliferation [25]. After successfully restoring miRNA-34a activity in LLC and 4T1 cells via lipofectamine-based miRNA-34a overexpression or using the designed ELP formulations, we confirmed the antiproliferative effects of miRNA-34a through cell viability assays (Fig. 6b, d). Cell proliferation was remarkably inhibited using varying treatment concentrations in miRNA-34a/lipofectamine (miR‐34a/lipo) and miR‐34a/ELP transfected cells compared to NC miRNA‐lipofectamine or NC miRNA/ELPs formulation alone. Notably, the complexation of tumor-suppressive miRNA-34a with Tat-A86 improved its cytotoxicity in LLC and 4T1 cells, surpassing the cytotoxicity achieved with Tat-E60 and A86. Especially, treating 4T1 cells with miRNA-34a complexed with Tat-A86 resulted in heightened efficacy and, consequently, a significant reduction in colony formation compared to that achieved using miRNA-34a complexed with Tat-E60 or A86 (Additional file 4: Figure S4). This underscores the pronounced efficacy of miRNA/Tat-A86 complexes, emphasizing their potential as a formidable platform for targeted cancer therapy.

Furthermore, we assessed the impact of increased miRNA‑34a expression facilitated by lipofectamine and ELP nanoparticles on the cell cycle. miRNA-34a targets anti-apoptotic proteins such as Bcl-2 and survivin, fostering augmented apoptosis and contributing to cell cycle arrest [11, 51]. Given the close interconnection between apoptosis and cell cycle progression, the regulatory role of miRNA-34a in apoptosis can intricately influence cell cycle dynamics. The upregulation of miRNA-34a via lipofectamine induced a discernible G1 phase cell cycle arrest, while the miRNA‐34a/ELP formulations arrested the cell cycle at the G2/M phase (Fig. 7a, c). Intriguingly, Tat-A86 exhibited superior cellular accumulation in both G2 and M phases compared to the other experimental groups. The G2/M DNA damage checkpoint, a critical regulatory point in the cell cycle, ensures that cells refrain from initiating mitosis until any damaged or incompletely replicated DNA is adequately repaired post-replication [52]. Cells harbouring a defective G2/M checkpoint proceed into mitosis without proper DNA repair, leading to apoptosis or cell demise post-division [53]. In our study, exposure to miRNA‐34a/ELPs resulted in cell cycle arrest specifically in the G2/M phase, suggesting potential apoptosis induction. Consequently, treatment of LLC cells with the miRNA-34a/Tat-A86 complex induced substantial cell apoptosis compared to the miRNA-34a/Tat-E60 or miRNA-34a/A86 complexes. The heightened cell apoptosis observed in miRNA-34a/Tat-A86 complexes may be attributed to stable complexation with miRNA-34a, improved uptake efficiency, and successful miRNA-34a release (Fig. 7b, d). These factors collectively contribute to inhibiting cell cycle progression in the LLC cells.

Targeted miRNA delivery is an effective strategy in cancer therapy because it allows for selectively influencing cancer cells while sparing normal cells. In vitro assessments definitively illustrated the pronounced efficacy of the miRNA/ELP formulations in facilitating miRNA delivery into LLC cells. However, before conducting the in vivo tumor inhibition study, the efficacy of FAM-miRNA delivery using diverse ELP formulations was assessed using an in vitro 3D model. Notably, Tat-A86 facilitated deeper penetration of FAM-miRNA, in contrast to the restricted penetration observed with Tat-E60 or A86, which remained confined to the outermost cellular layers of the spheroids at 4 h (Additional file 7: Figure S7). The mean intensity of FAM-miRNA/Tat-A86 within LLC spheroids consistently surpassed that of FAM-miRNA-loaded ELPs across all treatment durations. This observation underscores the discernibly superior spheroid-penetrating capability of Tat-A86 compared to the other formulations.

Next, we optimized therapeutic regimens utilizing 3D spheroids of LLC cells before progressing to in vivo studies. Dose–response analyses, encompassing a range of miRNA-34a concentrations (5 μM, and 10 μM), were conducted within the 3D model to gain insights into the potential in vivo efficacy of miRNA-based therapies. The results revealed that LLC spheroids treated with either fresh culture medium or ELPs alone exhibited no discernible inhibition of spheroid growth over a 10-day period (Additional file 8: Figure S8a–c). The spheroid diameter increased over time, with the cells becoming densely compacted due to intercellular interactions and extracellular matrix (ECM) secretion, indicative of cell proliferation in the outer layers of the spheroids (Fig. 8a–d). In contrast, treatment with miRNA-34a/Tat-A86 significantly inhibited spheroid growth for 6 d (Fig. 8c, d). By day 10, the spheroid diameter decreased markedly, suggesting that the outer layer cells were effectively eliminated due to the antitumor effects of miRNA-34a. Simultaneously, spheroid diameters in groups treated with miRNA-34a/Tat-E60 and miRNA-34a/A86 exhibited inhibition on day 10 post-treatment, probably because of the gradual release of miRNA-34a from the nanoparticles. Moreover, a significant reduction in spheroid diameter was observed at miRNA concentration of 10 μM. Overall, the results indicated that miRNA-34a-loaded Tat-A86 inhibited the growth of LLC spheroids more efficiently than the other ELPs.

After validating different therapeutic regimens with 3D spheroids of LLC cells, the therapeutic efficacy of Tat-A86-mediated miRNA-34a delivery was assessed in an allograft LLC model in C57BL/6 mice. Systemic intravenous administration of miRNA-34a/ ELPs effectively inhibited tumor growth compared to the PBS-treated control group (Fig. 9b). Thus, systemic administration of miRNA-34a alone had no discernible effect on LLC tumor growth. This lack of efficacy can be attributed to the inherent susceptibility of free miRNA-34a to degradation by nucleases, limiting its stability and half-life in circulation. In contrast, Tat-A86-mediated delivery of miRNA-34a resulted in enhanced antitumor effects. To elucidate the intrinsic advantages of various ELP nanoparticles, we deliberately used a suboptimal dose of miRNA-34a (6 mg/kg). The results revealed that the administration of miRNA-34a/Tat-A86 substantially augmented tumor growth inhibition by ~ 2.6-fold. This robust effect strongly supports the postulation of targeted delivery, specifically facilitated by Tat-A86 nanoparticles. Tumor volume and weight measurements at the end of the therapy revealed a statistically significant reduction in mean tumor weight in the miRNA-34a/Tat-A86 group (0.37 g) compared to both the miRNA/Tat-E60 group (0.56 g) and the untreated cohort (~ 0.94 g) (Fig. 9e). These findings unequivocally affirm the potent antitumor effect exerted by miRNA-34a/Tat-A86 nanoparticles in LLC cells. Remarkably, no discernible reductions in body weight or indications of tissue injury were observed before or after the therapeutic intervention. The sustained equilibrium in body weight throughout the treatment duration provides clear evidence that miRNA-34a/Tat-A86 elicited no overt signs of gross toxicity (Fig. 9c). Furthermore, H&E staining of major organs revealed no apparent tissue damage relative to the PBS-treated control group (Fig. 10). TUNEL staining of tumor tissues further validated the proapoptotic activity of the miRNA-34a/Tat-A86 nanoparticles (Fig. 11). Furthermore, a comprehensive analysis of the H&E-stained tumor sections suggested a notably heightened degree of cellular death in the miRNA-34a/Tat-A86 group compared to the respective control groups.

Overall, the Tat-A86 nanoparticle formulation for miRNA-based cancer therapy is a highly promising candidate for clinical application, primarily due to its favorable toxicity profile. Additionally, the Tat-A86 nanoparticle showcases an impressive ability to selectively target tumor sites, thereby augmenting the therapeutic effectiveness of miRNA-based interventions. Delivering miRNA-34a through a unified formulation with precise targeting of tumor tissues constitutes a pivotal advantage in the current paradigm. Furthermore, the integration of Tat-A86 facilitates the comprehensive evaluation of miRNA-34a functionality in both in vitro experiments and animal models. Such outcomes contribute significantly to cancer research and advance the potential of miRNA-based therapies.

## Conclusion

This study explored the potential therapeutic application of a novel miRNA delivery system based on Tat-A86—an ELP-based nanoparticle. This approach holds promise for modulating miRNA expression and activity, offering new avenues for miRNA replacement therapy in cancer treatment. Tat integration into the A86 framework, which comprises multiple iterations of the IL-4R targeting peptide AP-1 along the ELP backbone, augments the efficiency of miRNA gene delivery to the cytosol. This augmentation, in turn, enhances the precision and efficacy of therapeutic interventions. After successfully synthesizing the miRNA-34a/Tat-A86 nanoformulation, its impact on LLC cells was systematically assessed both in vitro and in vivo. The miRNA-34a/Tat-A86 nanoformulation exhibited high suitability for miRNA-34a delivery to tumor sites by actively targeting IL-4R, a receptor highly expressed in most types of solid tumors. The ELP-based Tat-A86 nanoparticle adeptly condensed miRNA molecules into stable nanocomplexes under physiological conditions. Furthermore, the nanocomplexes showed ease in associating with cellular membranes and facilitated efficient cellular uptake, likely through endocytosis. These properties make it a promising formulation for improved miRNA delivery and cell uptake, contributing to potential anticancer properties. Additionally, miRNA-34a/Tat-A86 complexes induced maximum cell apoptosis compared to other ELPs, demonstrating successful intracellular miRNA-34a release facilitated by Tat-A86. In 3D LLC spheroids, Tat-A86-mediated miRNA-34a delivery resulted in superior penetrating ability compared to free miRNA, Tat-E60, or A86 nanoparticles. In vivo tumor inhibition examinations showed that Tat-A86 nanoparticles are more effective in impeding tumor growth compared to free miRNA and other formulations. This substantiates the conjecture that the Tat-A86 delivery system holds promise for broader applicability across diverse cancer types and with varied miRNAs. The comprehensive findings of this study underscore the proficient accomplishment of non-viral miRNA delivery using the novel ELP-based Tat-A86 nanoparticles, thereby unveiling prospective applications in precision-targeted cancer therapeutics.

### Supplementary Information

Additional file 1.

## Data Availability

All data generated or analyzed during this study are included in this published article.

## Abbreviations

CPP: Cell-penetrating peptide
ELP: Elastin-like polypeptide
IL-4R: Interleukin-4 receptor
ITC: Inverse transition cycling
Tt: Transition temperature
TEM: Transmission electron microscope
DLS: Dynamic light scattering
OD: Optical density
Da: Daltons
PBS: Phosphate buffer saline
LLC: Lewis lung carcinoma
ECM: Extracellular matrix
PI: Propidium iodide
H&E: Hematoxylin and eosin
TUNEL: Terminal deoxynucleotidyl transferase-mediated dUTP nick end labeling
