# Supplementary material for: Multifunctional elastin-like polypeptide nanocarriers for efficient miRNA delivery in cancer therapy
Source: J Nanobiotechnology. 2024 May 27;22:293. doi: 10.1186/s12951-024-02559-5 (PMC11131307; doi:10.1186/s12951-024-02559-5)
Supplement: Supplementary file 1 — Additional file 1. [file 12951_2024_2559_MOESM1_ESM.docx]

**Supplementary materials**

**Multifunctional elastin-like polypeptide nanocarriers for efficient miRNA**

**delivery in cancer therapy**

Jisan Hong ^1^, Dahye Sim ^1^, Byung-Heon Lee ^1^, Vijaya Sarangthem ^1,^*, Rang-Woon Park ^1,^*

^1^Department of Biochemistry and Cell Biology, Cell & Matrix Research Institute, Kyungpook National University, School of Medicine, Daegu 41944, Republic of Korea.

* Corresponding authors:

Rang-Woon Park, Ph. D, MD, Professor,

Department of Biochemistry and Cell Biology, Cell & Matrix Research Institute, Kyungpook National University, School of Medicine, Daegu 41944, Republic of Korea

E-mail address: [nwpark@knu.ac.kr](mailto:nwpark@knu.ac.kr)

Telephone No.: +82 53 420 4822; Fax: +82 53 422 1466

Vijaya Sarangthem, Ph. D

Department of Biochemistry and Cell Biology, Cell & Matrix Research Institute, Kyungpook National University, School of Medicine, Daegu 41944, Republic of Korea

E-mail address: [devi1703@gmail.com](mailto:devi1703@gmail.com) (V. Sarangthem)

**Clonogenic assay**

miRNA-34a/ELPs complexes induced toxicity was evaluated in terms of clonogenic inhibition. To assess colony formation, 4T1 cells were treated with variables complexes miRNA-34a/ELPs for 1 h and seeded into 6-well plates and the cells were then observed for colony formation after 14 days. The cells were then fixed with methanol, stained with 0.5% crystal violet for 20 min, and visible colonies were counted under a microscope (Leica TE2000-S microscope, Tokyo, Japan). The colonies formation was analysed in compared with negative control (NC) miRNA/ELP complex treated group. As shown in Figure S4 Tat-E60/miRNA-34a and A86/miRNA-34a have shown minimal colonies reduction by ~ 83.7% or 82.2%. Comparatively, Tat-A86 complexes demonstrated a more effective inhibitory effect, resulting in a 48.8% reduction in colony formation.

**Uptake assay of miRNA-34a/ELPs**

Spheroid cultures of LLC cells were established in U-bottom clear 96-well plates featuring a cell-repellent surface. Each well was seeded with 10,000 LLC cells in 200 μl of culture medium, and spheroid formation was initiated through centrifugation at 1000g for 10 min. These plates were then incubated under standard cell culture conditions, at 37°C with 5% CO_2_ in humidified incubators, allowing the cells to grow for 72 h until reaching multicellular tumor spheroids with an approximate diameter of 400 μm. To assess the penetration capability of the ELP nanoparticles, the spheroids were treated with FAM-labeled NC-miRNA complexes, formed by combining miRNA at a concentration of 400 nM with their respective ELPs. After 1 h or 4 h incubation, the tumor spheroids were fixed with 4% paraformaldehyde, and the permeability of these nanoparticles was analyzed using confocal microscopy.

**Cell dependent LLC spheroid generation**

Spheroid cultures of LLC cells were established in 96-well U-bottom plates with a cell-repellent surface. Cells were seeded at three distinct concentrations: 5000, 10000, and 15000 cells per micro-well, and cultured for 12 days. To monitor spheroid growth over time, approximately 36 spheroids for each cell seeding concentration were imaged daily using light microscopy (Nikon, Tokyo, Japan). The spheroid diameter at various time points was measured using ImageJ (NIH, Bethesda, MD, USA). Simultaneously, the viability of LLC spheroids was assessed using a Live/Dead Viability/Cytotoxicity Kit from Biotium, USA. This assay allowed for a visual determination of whether cells within the spheroids remained viable after spheroid formation. At day 3, day 6, and day 10, spheroids of different sizes were incubated with a solution containing 1 μM calcein AM and 4 μM ethidium homodimer-1 at 37 °C for 1 h. Fluorescence microscopy (Nikon, Japan, model TS100-F) was employed to capture images using laser excitation of the sample at 488 nm and 561 nm.


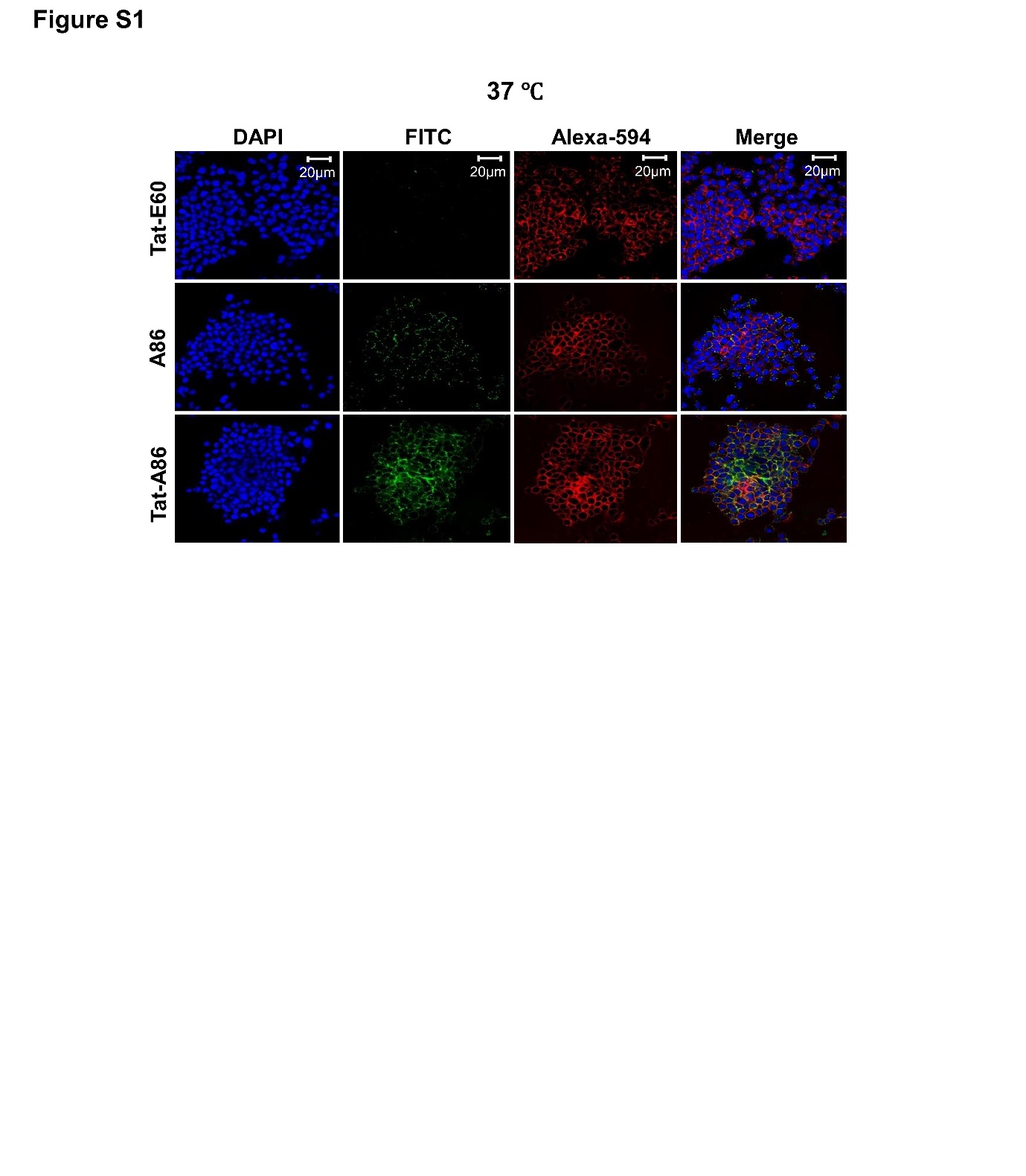


**Figure S1.** LLC cells were incubated with 0.3125 µM concentrations of the corresponding fluorescein isothiocyanate **(**FITC)-labelled ELPs (Tat E60, A86, and Tat-A86) at 37°C for 1 h, followed by fixation with 4% paraformaldehyde (PFA). Subsequently, cell membranes and nuclei were stained with Wheat Germ Agglutinin Alexa 594 and Hoechst, respectively. The samples were then examined using confocal laser microscopy. Representative confocal images are shown from five independent experiments. Scale bar, 20 µm.


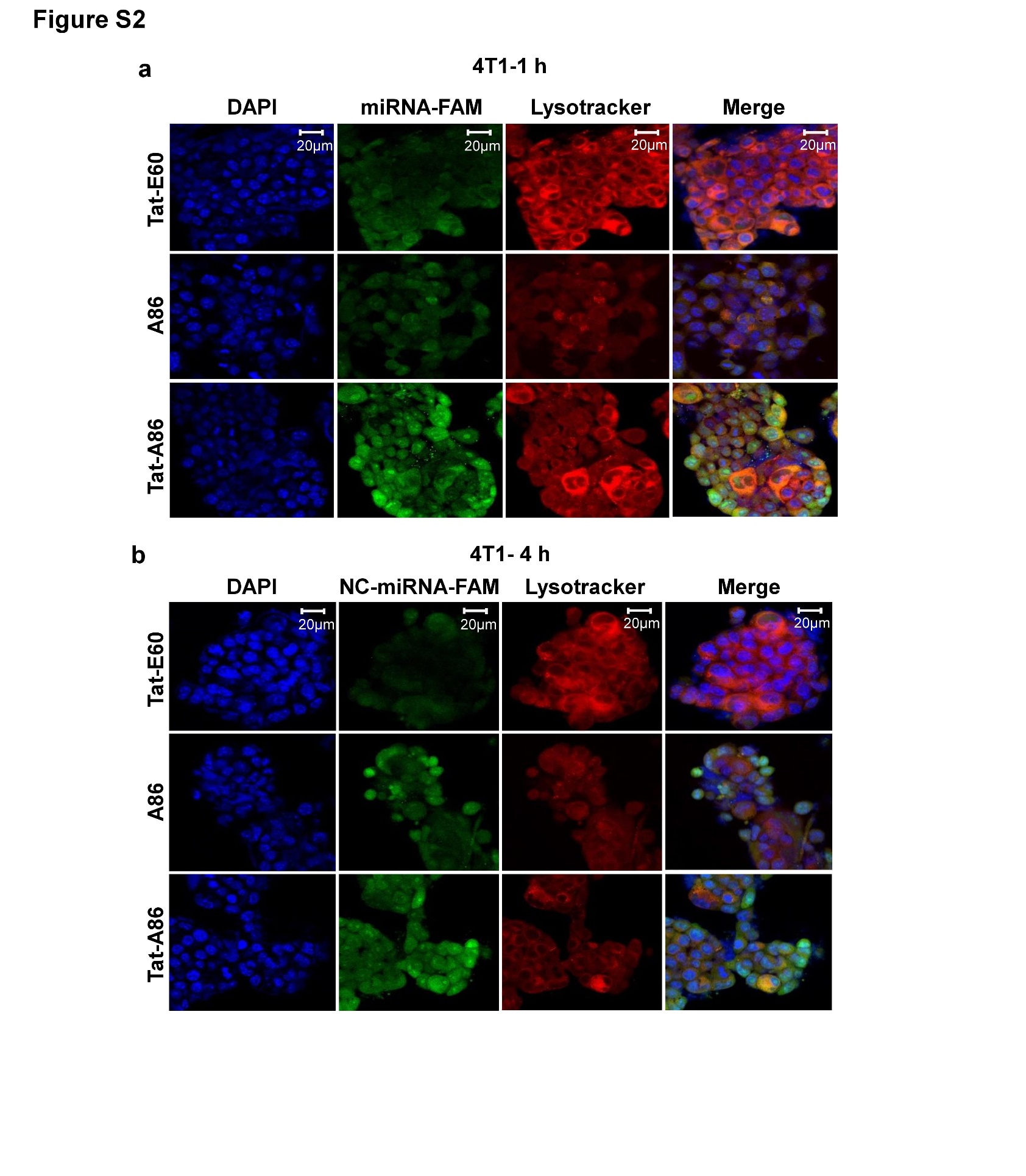


**Figure S2. Intracellular tracking of miRNA/ELP complexes**. 4T1 cells were incubated with miRNA/ELP complexes containing 150 pmol of FAM-labelled miRNA for 1 h (a) and 4 h (b). The subcellular localization of miRNA was examined using a Zeiss confocal microscope. Yellow signal represents the co-localization of miRNA with the lysosomal marker. Representative confocal microscopic images from five experiments (n=3) are shown. Blue indicates nuclei stained with Hoechst; Green represents miRNA/ELPs complexes; Red indicates LysoTracker. Scale bar, 20 µm.


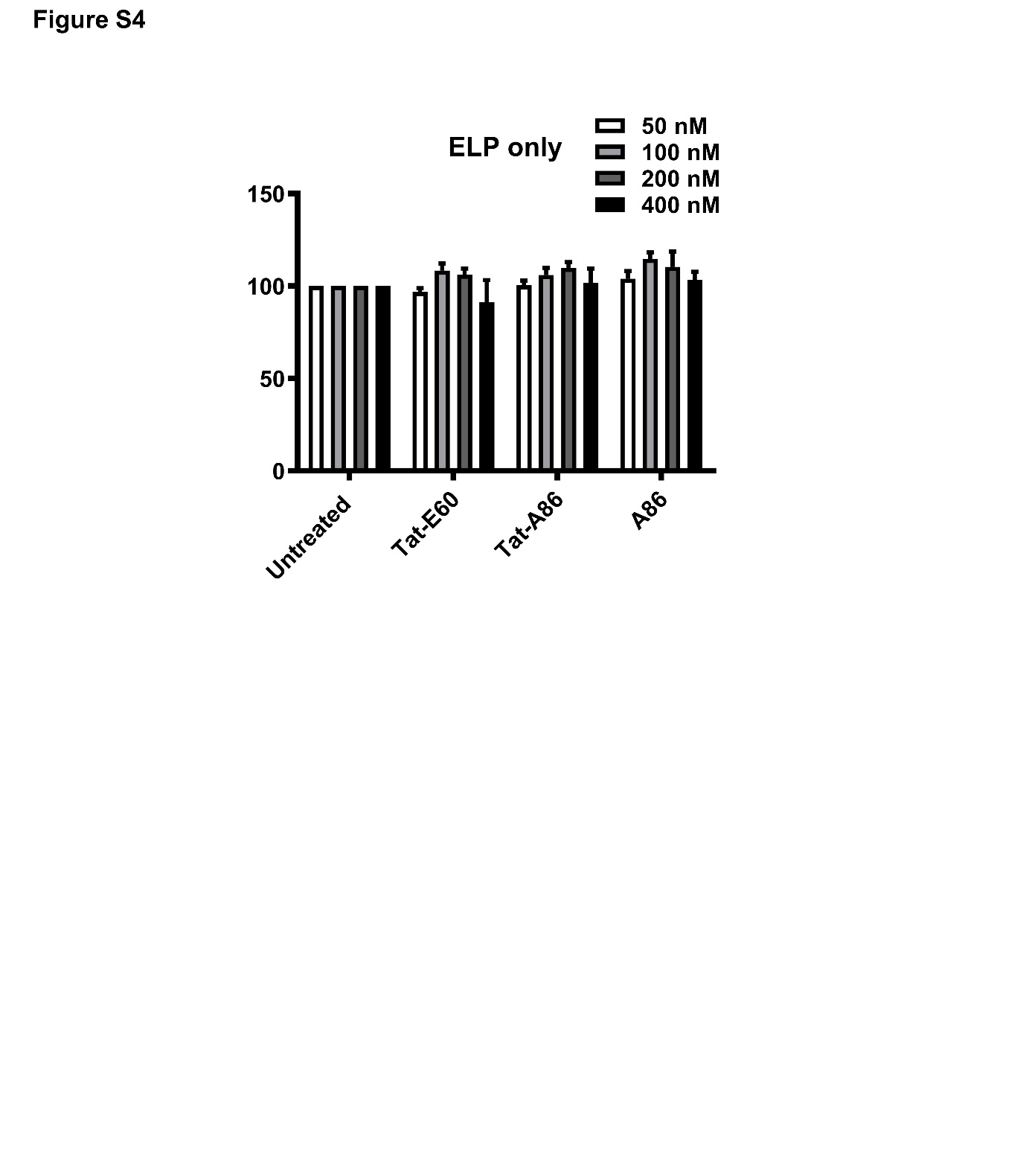


**Figure S3. Cell viability assay.** To assess the impact of ELPs on 4T1 cell growth, (5 × 10^3^/well) cells were seeded per well in a 96-well plate. The cells were treated with ELPs at concentrations ranging from 1 to 8 μM, consistent with the concentrations used for miRNA encapsulation (50-400 nM), for 48 h. Subsequently, 10 μL of CCK-8 solution was added to each well and incubated for 1 h at 37 ℃. The change in absorbance for the CCK-8 reagent was detected at a wavelength of 450 nm.


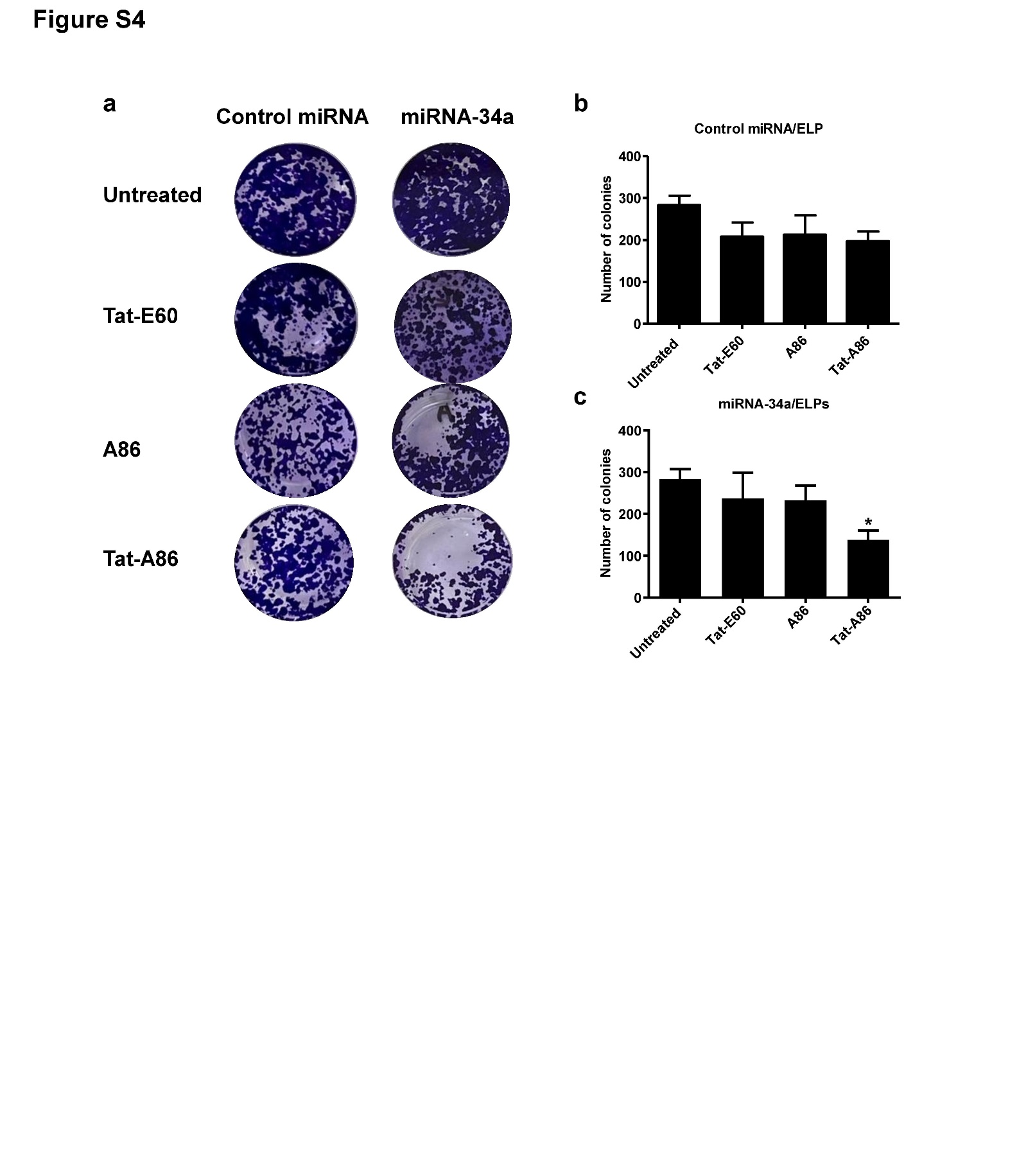


**Figure S4. Colony formation assay**. (a) 4T1 cells were treated with miRNA-34a/Tat-E60, miRNA-34a/A86, and miRNA-34a/Tat-A86 complexes, and subsequent colony formation was assessed. Plates were then stained with crystal violet, and colonies were counted using Image J. (b-c) Quantification of colony formation to determine the antitumor effect of miRNA-34a compared to the control NC miRNA. Data are presented as means ± S.D. (n=5). **p<0.01 (Tat-A86/miRNA versus untreated control).


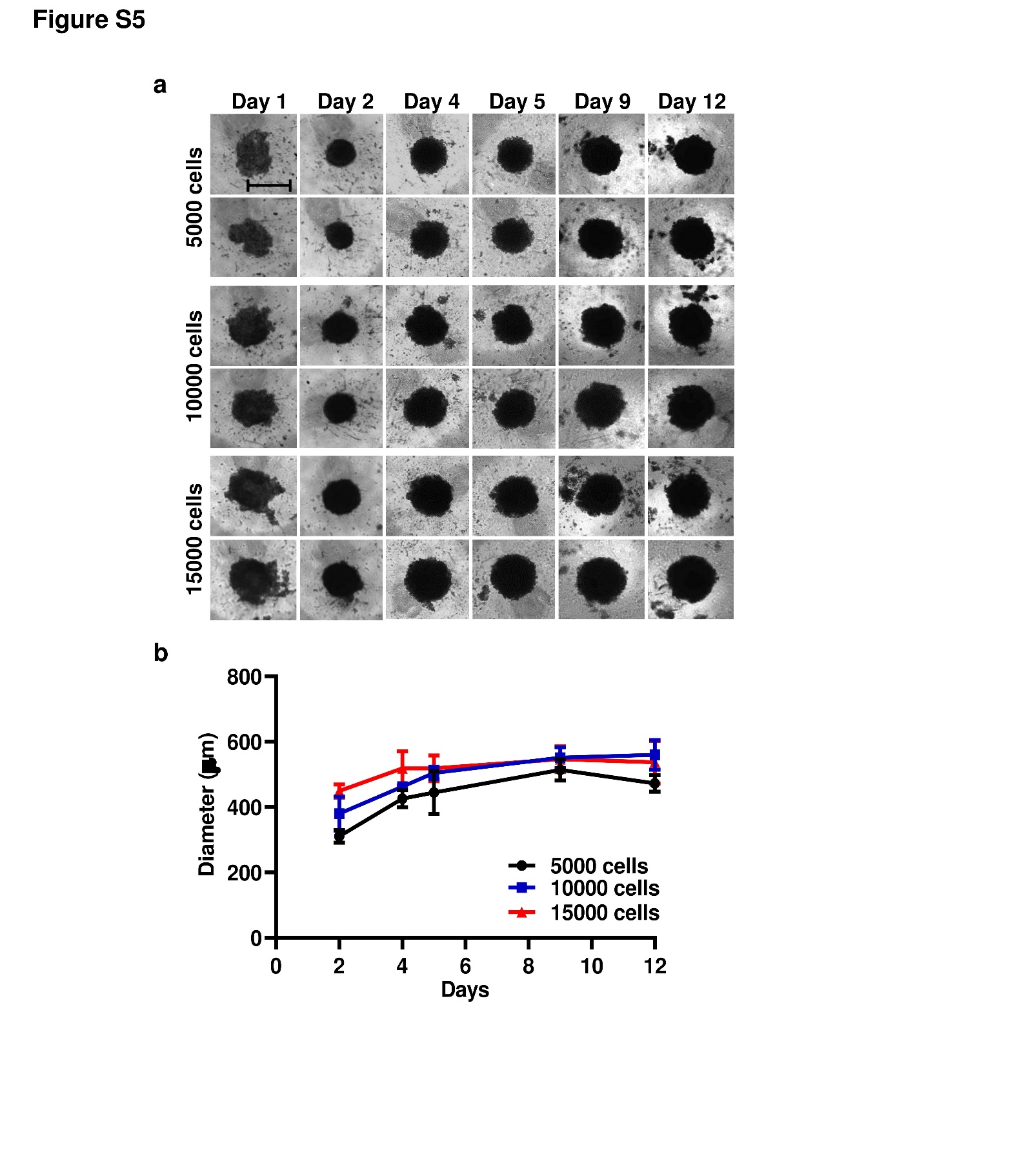


**Figure S5.** **LLC** **Spheroid growth characterization.** (a) LLC spheroids of different sizes were generated by adjusting the initial seeding cell concentrations. Spheroid diameters were determined over a 10-day culture period. Representative bright field images of one spheroids of distinct sizes. Scale bars, 500 μm. (b) Control of spheroid size by varying the cell seeding number. Data represent the mean ± standard deviation (SD) from a single experiment (n = 30).


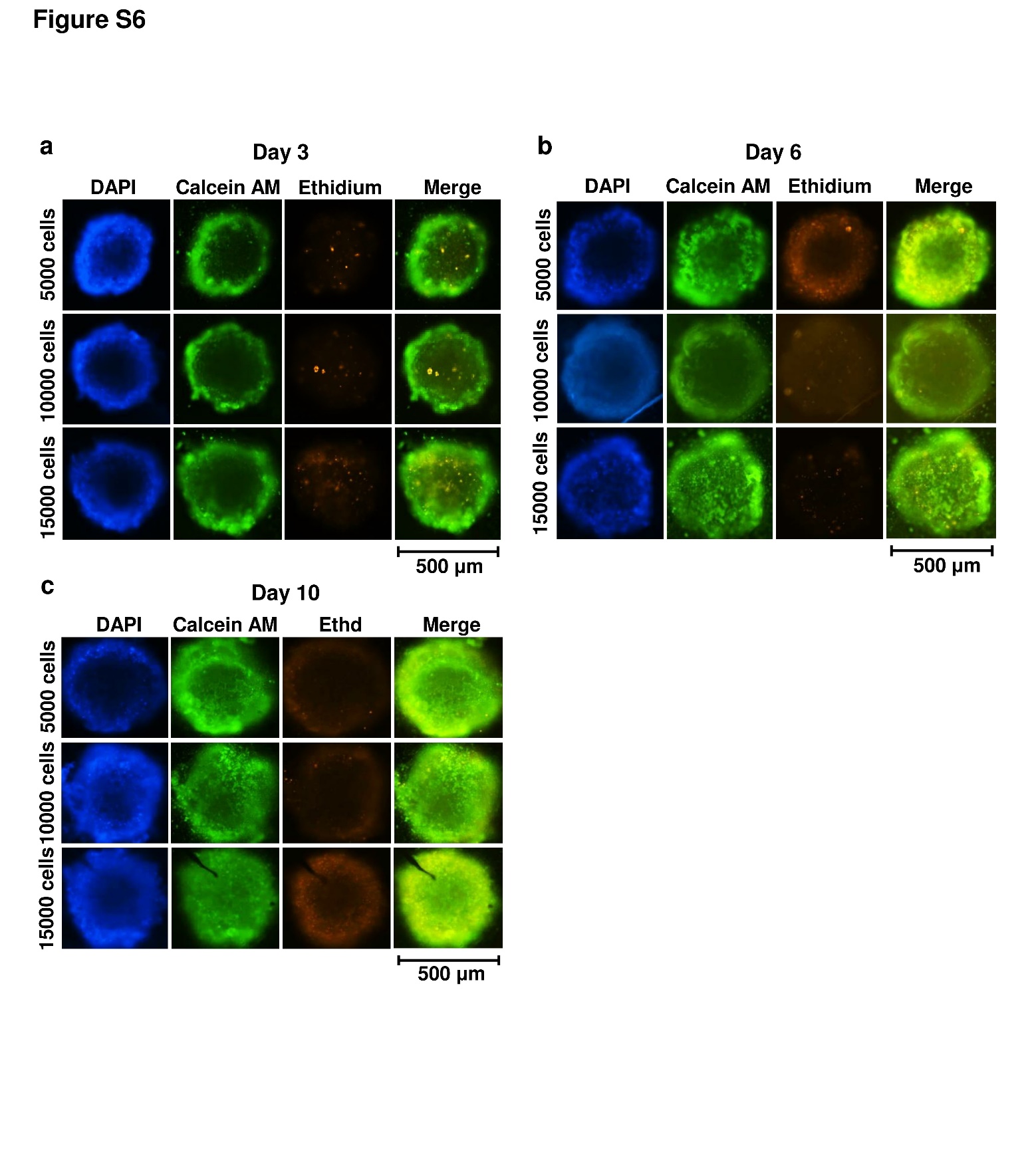


**Figure S6.** **Live and dead staining of LLC spheroid.** (a-c) Viability of the spheroids with different sizes was assessed using a live/dead staining with ethidium homodimer-1 and calcein AM. Viable cells appear as green, while nonviable cells appear as red. Scale bars, 500 μm.


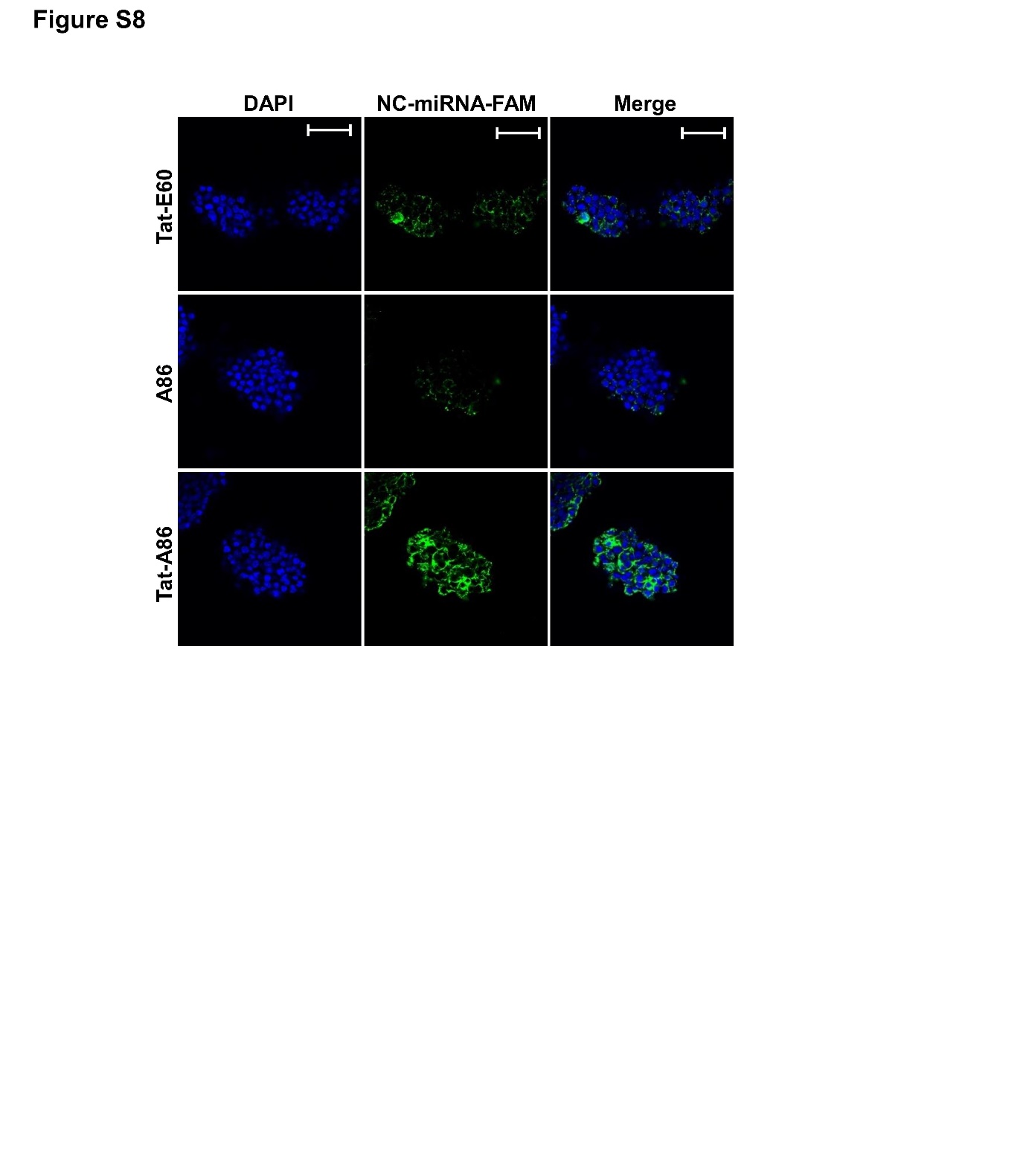


**Figure S7. ELP mediated miRNA uptake.** To evaluate the efficiency of ELPs in delivering the miRNA payload, 6-day-old LLC spheroids were treated with FAM-labeled NC-miRNA/ELPs complexes. Following a 1 h incubation, the tumor spheroids were fixed with 4% paraformaldehyde, and the permeability of these nanoparticles was analyzed using confocal microscopy. Scale bar = 100 μm.


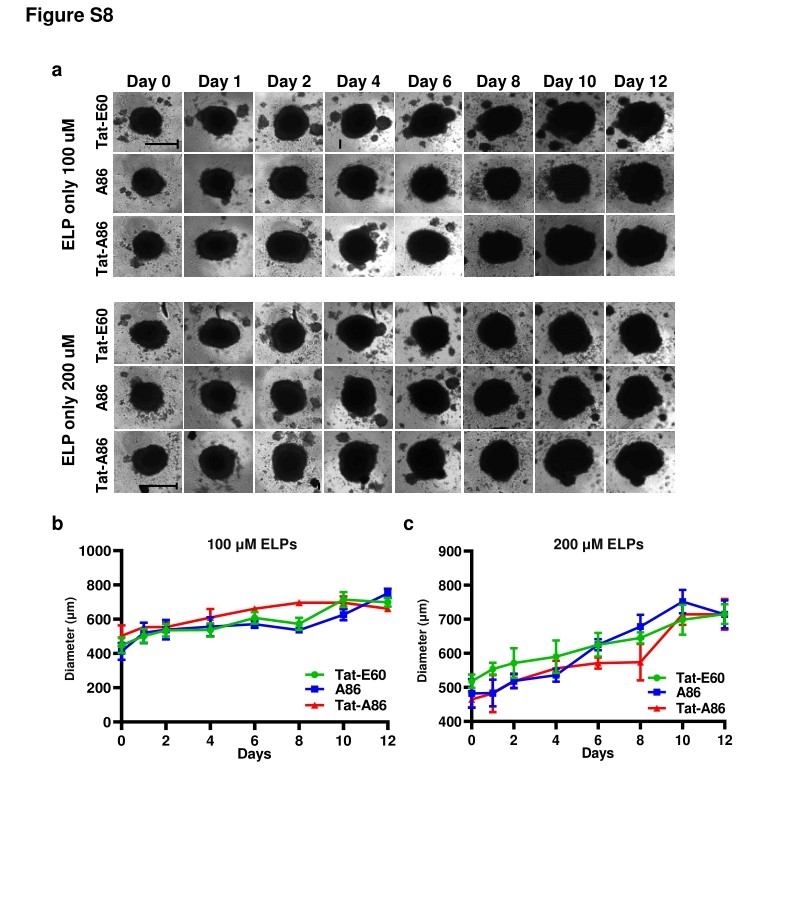


**Figure S8.**  Effect of ELPs on spheroid growth. (a) LLC spheroids were treated with ELPs concentration of 100 and 200 μM, featuring concentration used to encapsulate miRNA-34a concentrations of 5 or 10 μM. The time-dependent increase in diameter size of the LLC spheroids was observed through bright-field imaging over 1-10 days of culture, (scale bar = 500 μm). Representative images of spheroids of different sizes. (b-c) Growth curve of LLC spheroids following incubation with different ELPs concentrations. Data represent the mean ± standard deviation (SD) of one experiment (n = 30).
